# Prophylactic Activity of Orally Administered FliD-Reactive Monoclonal SIgA Against *Campylobacter* Infection

**DOI:** 10.3389/fimmu.2020.01011

**Published:** 2020-06-09

**Authors:** Lisa Perruzza, Stefano Jaconi, Gloria Lombardo, Debora Pinna, Francesco Strati, Diego Morone, Frauke Seehusen, Yue Hu, Sakshi Bajoria, Jian Xiong, Ozan Selahattin Kumru, Sangeeta Bagai Joshi, David Bernard Volkin, Renato Piantanida, Fabio Benigni, Fabio Grassi, Davide Corti, Matteo Samuele Pizzuto

**Affiliations:** ^1^Faculty of Biomedical Sciences, Institute for Research in Biomedicine, Università della Svizzera Italiana (USI), Bellinzona, Switzerland; ^2^Humabs BioMed SA a Subsidiary of Vir Biotechnology Inc., Bellinzona, Switzerland; ^3^Laboratory for Animal Model Pathology (LAMP), Institute of Veterinary Pathology, Vetsuisse Faculty, University of Zurich, Zurich, Switzerland; ^4^Department of Pharmaceutical Chemistry, Vaccine Analytics and Formulation Center, University of Kansas, Lawrence, KS, United States; ^5^Department of Otolaryngology–Head and Neck Surgery, Ospedale Regionale di Lugano, Lugano, Switzerland

**Keywords:** secretory IgA, monoclonal antibodies, prophylaxis, *Campylobacter*, flagellar-capping protein, FliD

## Abstract

*Campylobacter* infection is one of the most common causes of bacterial gastroenteritis worldwide and a major global health threat due to the rapid development of antibiotic resistance. Currently, there are no vaccines approved to prevent campylobacteriosis, and rehydration is the main form of therapy. Secretory immunoglobulin A (SIgA) is the main antibody class found in mucous secretions, including human milk, and serves as the first line of defense for the gastrointestinal epithelium against enteric pathogens. In this study, we describe the prophylactic activity of orally delivered recombinant SIgA generated from two human monoclonal antibodies (CAA1 and CCG4) isolated for their reactivity against the flagellar-capping protein FliD, which is essential for bacteria motility and highly conserved across *Campylobacter* species associated with severe enteritis. In an immunocompetent weaned mouse model, a single oral administration of FliD-reactive SIgA CAA1 or CCG4 at 2 h before infection significantly enhances *Campylobacter* clearance at early stages post-infection, reducing the levels of inflammation markers associated with epithelial damage and polymorphonuclear (PMN) cells infiltration in the cecum lamina propria. Our data indicate that the prophylactic activity of CAA1 and CCG4 is not only dependent on the specificity to FliD but also on the use of the SIgA format, as the immunoglobulin G (IgG) versions of the same antibodies did not confer a comparable protective effect. Our work emphasizes the potential of FliD as a target for the development of vaccines and supports the concept that orally administered FliD-reactive SIgA can be developed to prevent or mitigate the severity of *Campylobacter* infections as well as the development of post-infection syndromes.

## Introduction

*Campylobacter* is an established cause of diarrhea worldwide and has recently been added to the WHO list of bacteria whose antibiotic resistance might pose a global threat to human health (1). *Campylobacter*'s epidemiology differs between high-income countries, where infections are sporadic, and low- and middle-income countries, in which the infection is common in early childhood and a major cause of life-threatening acute watery diarrhea in infants (2). Considered as a leading zoonosis, *Campylobacter* infection is mainly associated with the consumption of contaminated undercooked animal meat (poultry being the primary bacteria reservoir), water, or unpasteurized milk (3).

*Campylobacter jejuni* and *Campylobacter coli* are the major causes of *Campylobacter* enteritis in humans, and despite the clinical relevance of other emerging species (4), they will unlikely be eclipsed in terms of prevalence and magnitude of the disease. Campylobacteriosis typically results in an acute, gastrointestinal illness characterized by watery or bloody diarrhea, fever, weight loss, and cramps that last on average 6 days (3, 5). Although considered a self-limiting disease, the severe dehydration associated with *Campylobacter* enteritis represents a significant cause of death among newborns and children particularly in developing countries (6). Furthermore, *C. jejuni* infections has been consistently linked with the onset of autoimmune conditions such as Guillain–Barré syndrome (GBS) (7, 8) and inflammatory bowel disease (IBD) (9).

Despite a yet incomplete understanding of *Campylobacter* pathogenesis, flagellum-mediated motility is widely presumed pivotal for virulence and pathogenicity, as demonstrated in both experimental animal models and in human healthy volunteer studies (10). Nevertheless, flagellin (FlaA), the major constituent of the flagellum, is not highly conserved even within the same *C. jejuni* species, and its heavy glycosylation pattern varies greatly depending on the strain and growth phase (11, 12). In addition, a recombinant non-glycosylated form of *C. jejuni* flagellin was shown to be poorly immunogenic in clinical trials (2). Moreover, the possibility to use *C. jejuni* as vaccines has been limited by the risk of GBS development associated with ganglioside mimicry of bacterial lipo-oligosaccharide (LOS) (2).

Due to these shortcomings, there are currently no vaccines approved to prevent *Campylobacter* infection. Rehydration is the main form of therapy, and albeit antibiotics have been shown to be beneficial in severe infections, they are often not recommended due to the rapid development of antibiotic resistance (1). Despite recovering from infection, continuous exposure of infants in low-income countries to intestinal pathogens, including *Campylobacter*, has been linked to environmental enteropathy (EE)/environmental enteric dysfunction (EED), a subclinical chronic inflammation of the small intestine. This inflammation is associated with malabsorption of nutrients, growth faltering, impaired cognitive development, changes in microbiota, and reduced responsiveness to oral vaccination (13).

Secretory immunoglobulin A (SIgA) represents the most abundant class of antibodies produced in humans and serves as the first line of defense at the level of intestinal mucosa against enteric toxins and pathogens (14). Typically, a SIgA consists of two monomeric IgA linked tail to tail by disulfide bonds via a 15-kDa protein called joining (J) chain and further complexed with a heavily glycosylated secretory component (SC) (15). While IgA monomers and the J chain are synthesized, assembled, and secreted by plasma cells in the lamina propria, the SC consists of the ectodomain of the polymeric immunoglobulin receptor (pIgR) that remains bound to polymeric IgA (pIgA) following transcytosis across the enterocyte (16). The SC protects the antibody from proteolytic degradation, confers innate recognition functions, and contributes to appropriate tissue localization by anchoring the immunoglobulin to the mucus lining the epithelial surface (17, 18). SIgA acts primarily by preventing adhesion of pathogens to epithelial cells through receptor blockade, steric hindrance, and/or immune exclusion, eventually facilitating their clearance from the intestinal lumen via peristaltic and mucociliary activities (14, 19). Furthermore, antigen-bound SIgA can be transported back into Peyer's patches (PPs) subepithelial dome (SED) by microfold (M) cells in a process known as reverse transcytosis, which promotes migration and activation of follicular DCs, thus conditioning the inflammatory response associated to infection by enteric pathogens (14, 20). SIgA is also the main antibody class found in mucous secretions, including human milk, in which the Ig portion of the molecule is produced by mammary gland plasma cells that originate in the small intestine (entero-mammary pathway) (21). The provision of SIgA by breastfeeding has been reported to be critical for the protection against enteric pathogens especially in early infancy, when the contribution of endogenous production is limited (22–24).

In this study, an immunocompetent mouse model of *Campylobacter* infection was used to evaluated the prophylactic activity of orally delivered recombinant SIgA generated from human monoclonal antibodies isolated and selected for their reactivity against the flagellar-capping protein FliD, which is pivotal for bacteria motility (25), and highly conserved across *Campylobacter* species frequently associated with severe neonatal enteritis (26). We demonstrated that, regardless of the IgA isotype, prophylactically administered FliD-reactive SIgA can enhance *Campylobacter* clearance at early stages post-infection, dramatically reducing the levels of inflammation markers associated with epithelial damage and polymorphonuclear (PMN) cells infiltration. Our data indicate that the prophylactic activity of these antibodies is not only dependent on the specificity to FliD but also on features strictly associated with the SIgA format as the IgG versions of the same antibodies did not produce a comparable beneficial effect.

## Materials and Methods

### Production of Recombinant FliD Antigen

FliD amino acid sequences from *C. jejuni* (*n* = 38) and *C. coli* (*n* = 17) isolates were retrieved from GenBank and aligned using MEGA software to obtain a consensus sequence. Condon optimization and synthesis of the corresponding nucleotide sequence was outsourced (Genscript), and the construct was subcloned in pET21a in frame with the C-term His tag. The plasmid was transformed to *Escherichia coli* Rosetta strain. Gene expression was induced by the addition of 1 mM isopropyl-beta-d- thiogalactopyranoside (IPTG) to log-phase grown bacteria. After 5 h of induction at 37°C, bacteria were collected by centrifugation and stored at −20°C for 16 h before being resuspended in sonication buffer (50 mM NaH_2_PO_4_, 300 mM NaCl, 10 mM imidazole, and pH 7.4) and being sonicated 4 × 30 s in ice. Lysed bacteria were spun (14,000 × *g*, 1 h, and 4°C), and the supernatants were filtered and then loaded on a nickel column (HiTrap Chelating HP, GE HealthCare). Column was washed with washing buffer (50 mM NaH_2_PO_4_, 300 mM NaCl, 10 mM imidazole, and pH 7.4) followed by washing buffer + 0.1% Triton-X114 and washing buffer again. Bound protein was eluted from the column with elution buffer (50 mM NaH_2_PO_4_, 300 mM NaCl, 500 mM imidazole, and pH 7.4) and dialyzed for 16 h at 4°C in Dulbecco's phosphate-buffered saline (DPBS). Protein size was measure on size exclusion column (HiLoad 16/600 Superdex 75 pg; GE Healthcare), and the concentration was measured before storage at −20°C.

### Isolation of FliD-Reactive Memory B Cells and Generation of Recombinant mAbs

FliD-reactive monoclonal antibodies CAA1 and CCG4 were isolated, as previously described (27, 28), from IgA^+^ and IgG^+^ memory B cells derived from tonsillar donors who had given written informed consent, following approval by the Cantonal Ethical Committee of Cantone Ticino, Switzerland (CE3536 2019-02061). The sequences for the variable regions of the mAbs were obtained from memory B cell clone messenger RNAs (mRNAs) following reverse transcription PCR (RT-PCR) and sequencing. The VH region of each mAb was cloned into a plasmid encoding IgA1, IgA2 allotype m(2) (29), or IgG1 heavy chains. The constructs were transiently transfected in Expi293 cells together with those for the corresponding light chains and, in the case of SIgA, also with those encoding for the joining chain (J) and the ectodomain of pIgR (SC) (29). After 48 h, cells were supplemented with supplement medium and further incubated for 5 days under shaking. Next, cells were spun down, and the supernatant was filtered before being purified using CaptureSelect™ IgA Affinity Matrix (Thermo Fisher). SIgA assembly was appraised by ultrahigh performance liquid (UHPL) chromatography using ACQUITY BEH SEC Guard Column (Waters) and by denaturing non-reducing gel using SIgA purified from human milk (MP Biomedicals) as positive control. The purified immunoglobulins were quantified with Pierce™ Rapid Gold BCA Protein Assay Kit (Life Technologies) and then aliquoted and store at −80°C.

### N-Glycan Oligosaccharide and Total Carbohydrate Analysis

N-Glycan oligosaccharide analysis was performed as previously described (30). Briefly, a GlycoWorks RapiFluor-MS N-Glycan Kit (Waters Corporation, Milford, MA) was used to identify and quantify N-linked glycans following the manufacturer's instructions. Fluor-MS N-glycan analysis was performed using an Agilent 1,260 Infinity II HPLC system equipped with a 1,260 FLD detector (Agilent, Santa Clara, CA) and an Agilent 6,230 electrospray ionization time-of-flight mass spectrometer (Agilent, Santa Clara, CA). A HILIC AdvanceBio Glycan Mapping column (120 Å, 2.1 × 150 mm, 2.7 μm), operated at 45°C, was used to separate various N-glycans. Fifty microliters of prepared samples was injected into liquid chromatography–mass spectrometry (LC-MS) system, with a flowrate of 0.6 mg/ml and a gradient run time of 55 min. Fluorescence was obtained using excitation and emission wavelengths of 265 and 425 nm, respectively. MS was acquired simultaneously from 400 to 2,000 *m*/*z* at a constant scan rate of one spectrum per second. N-Glycans were assigned based on *m*/*z* values using the N-glycan Glycobase database, and N-glycan quantification was calculated on integration of the fluorescence chromatogram.

Total carbohydrate analysis was performed as previously described (30). Briefly, a glycoprotein carbohydrate estimation kit (Thermo-Fisher #23260) was used to determine the total carbohydrate content (both N- and O-linked oligosaccharides) in mAb samples as a percentage of total protein mass. Lysozyme, bovine serum albumin (BSA), ovalbumin, Apo-transferrin, fetuin, and α1-acid glycoprotein were used as glycoprotein standards to construct a standard curve.

### Size Exclusion Chromatography and Sedimentation Velocity Analytical Ultracentrifugation

Size exclusion chromatography (SEC) was performed as previously described (30). Briefly, a Shimadzu Prominence ultrafast liquid chromatography HPLC system equipped with a diode array detector (with absorbance detection at 214 nm) was equilibrated at 0.5 ml/min flowrate in 0.2 M sodium phosphate buffer, pH 6.8 for at least 2 h. Ten microliters of each mAb (10 μg total protein) was injected and separated by a TOSOH TSKgel G4000SWXL column (8 μm particle size, 7.8 mm ID × 30 cm) with the corresponding guard column operated at ambient temperature (Tosoh Biosciences) using a 30-min run time. Gel filtration molecular weight standards (Bio-Rad, Hercules, CA) were injected before and after the mAb samples to ensure integrity of the column and HPLC system. Potential presence of larger aggregates was determined by running mAb samples with and without the SEC column (i.e., protein percentage recovery). Data were analyzed using LC-Solution software (Shimadzu, Kyoto, Japan).

Sedimentation velocity analytical ultracentrifugation (SV-AUC) was performed as described previously (30) using a Proteome Lab XL-I (Beckman Coulter) analytical ultracentrifuge equipped with a scanning ultraviolet–visible optical system. All experiments were performed at 20°C after at least 1 h of equilibration after the rotor reached 20°C. SV-AUC was performed at a rotor speed of 40,000 rpm and with detection at 280 nm. The data were analyzed using Sedfit software (Dr. Peter Schuck, NIH).

### Intrinsic Fluorescence Spectroscopy and Relative Solubility Measurements

Intrinsic tryptophan fluorescence spectroscopy measurements as a function of temperature and polyethylene glycol (PEG) relative solubility measurements were both performed as described previously (30). For fluorescence spectroscopy measurements, 20 μl of each mAb sample was diluted to 0.2 mg/ml in PBS, pH 7.2 and loaded into a 384-well plate (Hard-Shell 384-well PCR plates) and overlaid with 2 μl of silicon oil (Thermo Fisher Scientific, Waltham, MA). Samples were excited at 295 nm, and the emission spectra were recorded from 300 to 450 nm with an integration time of 100 ms using a fluorescence plate reader equipped with a charge-coupled device (CCD) detector (Fluorescence Innovations, Minneapolis, MN). Temperature ramps were programmed from 10 to 100°C with an increment of 2.5°C per step. The mean center of spectra mass peak algorithm was used to analyze the data to determine the shift in fluorescence peak position as a function of temperature. With respect to relative solubility measurements, various concentrations of PEG 10,000 solutions ranging from 0 to 25% *w*/*v* were prepared with a final mAb concentration of 0.2 mg/ml. Samples were incubated overnight at room temperature in the dark in 384-well plates with 30 μl in each well. The next day, the plates were centrifuged for 15 min at ~1,200 × *g*, and the supernatant was transferred to a clean 384-well plate. Relative protein concentration in each well was then measured at 214 nm using a Spectramax M5 plate reader (Molecular devices). Thermal melting temperature values (*T*_m_) and % PEG midpoint calculations were performed using Origin v 9.1 software (OriginLab, Northampton, MA).

### Binding to Recombinant FliD: ELISA, Western Blot, and BLI Epitope Binning

Quantification of recombinant SIgA and IgG by ELISA was performed by coating 96-well plates with goat anti-human IgA or IgG (SouthernBiotech). Two-fold serial dilutions of the mAbs in duplicates were incubated for 1 h at room temperature. In the case of SIgA, detection was performed using human pIgR biotinylated antibody biotinylated anti-human SC antibody (R&D System) followed by incubation with streptavidin-AP, while goat anti-human IgG-AP (SouthernBiotech) was used for IgG. SIgA purified from human milk (MP Biomedicals) and Rituximab (MabThera) were used as quantification standards for SIgA and IgG, respectively.

Binding of CAA1, CCG4 SIgA, and IgG counterparts to the recombinant antigen was measured by ELISA using FliD-coated 96-well-plates following the same detection protocol described above.

Epitope binning was tested by biolayer interferometry (BLI) using Octet Red96 (ForteBio) immobilizing recombinant FliD antigen (5 μg/ml) onto APS Biosensor (Pall ForteBio) for 7 min. After incubation with blocking buffer (BB), the sensor was incubated with CAA1 SIgA2 (7.5 μg/ml) for 7 min before being further incubated with CCG4 SIgA2, CAA1 SIgA2, or BB for additional 7 min.

Binding of SIgA2 to FliD antigen in denaturing and reducing conditions was tested by Western blot. Briefly, 5 μg of recombinant FliD was loaded on sodium dodecyl sulfate–polyacrylamide gel electrophoresis (SDS-PAGE) NuPAGE 12% Bis–Tris protein gel (Thermo Fisher Scientific) in the presence of a reducing agent. Blotting was performed using IBLOT SYSTEM (Invitrogen), and the membrane was blocked with 5% milk (Biorad) in Tris-buffered saline with Tween 20 (TBST) for 1 h at room temperature (RT). The mAbs were biotinylated by incubation with 20 mM biotin using the EZ-Link NHS-PEO Solid Phase Biotinylation Kit followed by desalting with Zeba Spin Desalting Columns (Thermo Scientific). The membrane was incubated with a 1% blocking grade blocker (BioRad) in TBST containing 20 μg/ml of biotinylated mAbs followed by washes in TBST and by incubation with streptavidin-horseradish peroxidase (HRP). Detection was performed using ECL detection kit (GE Healthcare) on Fusion Fx7 (Witec AG).

Sensitivity to IgAse was evaluate by incubating CAA1 SIgA1 and SIgA2 with recombinant IgA protease from *Neisseria gonorrhoeae* (IgAse Pro-Pro-Y-Pro- Mo Bi Tec). Briefly, 100 μg of CAA1 SIgA1 and SIgA2 were incubated at 37°C for 18 h with 1.5 μg of IgAse or with reaction buffer alone. The post-incubation products were visualized in a denaturing non-reducing gel with HiMark Prestained Protein Standard (Thermo Fisher).

### Bacterial Strains and Growth Conditions

*C. jejuni* 81-176 and *C. coli* NCTC 11437 strains were purchased from the American Type Culture Collection (ATCC) and Public Health England bacteria collection, respectively. The *Campylobacter* species were grown from glycerol stocks in Mueller–Hinton agar (Sigma Aldrich) (15 g/L) supplemented with vancomycin and trimethoprim (10 μg/ml) (Sigma-Aldrich) for 96 h at 40°C in microaerophilic jar with Oxoid™ CampyGen™ 2.5 L Sachet (Fisher Scientific).

### Determination of mAb Binding to *Campylobacter* Flagellar-Capping Protein by Flow Cytometry and Microscopy

*In vitro* binding of the different FliD-reactive SIgA to a pure culture of *C. jejuni* was measured by flow cytometry. Fifty microliters of each mAbs (20 μg/ml) was incubated with 50 μl of *C. jejuni* 81-176 (10^7^ CFU/ml) for 1 h at 4°C. After washing with PBS 2% fetal bovine serum (FBS), bacteria were incubated for 30 min with biotin-conjugated anti-human IgA (Southern Biotech, Cat. No. 2050-08). Immunoglobulin-bound bacteria were then stained with streptavidin-PB at 4°C for 30 min. Samples were washed with PBS 2% FBS, resuspended in 2% paraformaldehyde and acquired on LSR Fortessa flow cytometer (BD Biosciences, Franklin Lakes NJ, USA) using forward scatter (FCS) and side scatter (SSC) parameters in logarithmic mode. Data were analyzed using the FlowJo software (TreeStar, Ashland, OR, USA) or FACS Diva software (BD Biosciences, Franklin Lakes NJ, USA).

Binding of CAA1 and CCG4 SIgA on the poles of *C. jejuni* was also confirmed by microscopy. Briefly, 30 μg of CAA1, CCG4, and HGN194 SIgA2 were incubated with 10^6^ CFU of *C. jejuni* 81-176 for 1 h at 37°C in microaerophilic conditions. Next, bacteria–mAbs complexes were washed and further incubated with antihuman IgA–AF647 conjugated (1:200, Jackson ImmunoResearch, Cat. No. 109-606-011) and SytoBC (1:1,000, Thermo Fisher Scientific, Cat. No. S34855) for additional 30 min before being poured in microscope slides. Images were acquired with a Leica SP5 laser scanning confocal microscope with a 488- and 633-nm excitation. The resulting fluorescence emission was collected using 490–627-nm (for SYTOX Green) and 636–770 nm (for Alexa 647) windows. Samples were imaged with a 63 × objective (N.A. 1.4) in *xy* optical sections of 61.51 μm (1,024 × 1,024 pixels) with a pixel size of 60.1 nm. To improve lateral resolution, the pinhole was set to 0.5 AU.

### Mice

Animal experiments were performed in accordance with the Swiss Federal Veterinary Office guidelines and authorized by the Cantonal Veterinary Office (TI-09-2019). Twenty-one-day-old C57BL/6 female mice were purchased from Charles River Laboratories. Mice were housed, five per cage, under standardized conditions (20 ± 2°C, 55 ± 8% relative humidity, 12 h light/dark cycle), at the Institute for Research in Biomedicine. Food and water were available *ad-libitum*, and mice were monitored daily.

### Analysis of mAbs-Specific Binding and Cross-Reactivity With the Murine Microbiota in Fecal Samples

In order to measure *C. jejuni* specific antibody binding to the mouse microbiota, stools were collected and homogenized in sterile PBS (0.01 g of stools in 100 μl PBS). Samples were centrifuged for 5 min at 400 g to pellet large debris, and then, the supernatant were centrifuged at 8,000 *g* for 5 min to pellet bacteria. The supernatant was removed, and the bacterial pellet was resuspended in 150 μl (50 μg/ml) of the different anti-Campylo mAbs and incubated at 4°C for 30 min. After washing, bacteria were incubated at 4°C for 30 min with biotin-conjugated anti-human IgA (Southern Biotech, Cat. No. 2050-08) and SYTO BC (1:1,000, Thermo Fisher Scientific, Cat. No. S34855). Bacteria were washed once and then incubated with streptavidin-PB and, at the end, resuspended in 2% paraformaldehyde in PBS for flowcytometry analysis. All the samples were acquired on an LSR Fortessa flow cytometer (BD Biosciences, Franklin Lakes NJ, USA) using FCS and SSC parameters in logarithmic mode. Data were analyzed using the FlowJo software (TreeStar, Ashland, OR, USA) or FACS Diva software (BD Biosciences, Franklin Lakes NJ, USA).

### mAbs Localization and Persistence in the Weaned Mice Intestinal Tract

Differences in localization and persistence of SIgA vs. IgG in the gastrointestinal (GI) tract of 21-day-old C57BL/6 mice were evaluated by testing the PK of the two species in the three GI subcompartments: small intestine, cecum, and colon. Weaned mice were administered with a PBS solution containing 150 μg of *Campylobacter*-irrelevant antibody HGN194, which targets HIV gp120 and presents no cross-reactivity with the murine microbiota, in the form of SIgA2m(2) or IgG. A control group was administered only with PBS. At 2, 4, and 8 h post-oral administration, five animals per group were euthanized, and the content of the three different intestinal tracts was collected and resuspended in 1.5 ml PBS. The samples were then homogenized, and after centrifugation, the supernatants were collected and stored at −20°C. The detection of human IgA or IgG in the different parts of the GI tract was performed by ELISA. Briefly, 96-well plates were coated with goat anti-human IgA or IgG antibodies (Southern Biotechnology: 2050-01 and 2040-01) and blocked with 1% BSA blocking buffer. Plates were then incubated with 2-fold serial dilutions (1:2 to 1:256) of each intestinal sample for 1 h at room temperature. After four washes with PBS + 0.05% Tween 20, the presence of the antibodies was detected by incubation with goat anti-human lambda antibody AP conjugated (Southern Biotechnology: 2070-04) for 1 h at room temperature followed by 15 min incubation with p-nitrophenylphosphate (pNPP) and detection at 405 nm. Taking into account the different numbers of lambda light chains possessed by the polymeric immunoglobulin, to quantify the amount of SIgA or IgG in the samples, two distinct standard curves were built using HGN194 SIgA and HGN194 IgG as standards, respectively. No signals were detected testing the samples from the control animals administered only with PBS.

### Oral Prophylaxis and Infection Protocol

C57BL/6 female mice (21 days old) were pre-treated with 10 mg of vancomycin (Sigma-Aldrich) in 200 μl PBS 48, 24, and 12 h prior to mAbs administration. Mice were orally administered with 200 μg of FliD-reactive mAbs in 200 μl PBS 2 h before being infected by oral gavage with *C. jejuni* 81-176 (10^8^ CFU/200 μl PBS). In order to monitor the prophylactic efficacy, mice were observed daily, and stools were collected at 24, 48, and 72 h post-infection. To monitor the pathogen load, stools were resuspended and plated on Mueller–Hinton agar plates containing 10 μg/ml of vancomycin and trimethoprim.

### Flow Cytometry Analysis of Cecum Lamina Propria PMNs

Mice were sacrificed 72 h post-infection; the cecum was removed, opened longitudinally, delicately separated by cecal content, and washed twice with ice-cold PBS. The cecum was digested at 37°C for 30 min with Roswell Park Memorial Institute (RPMI) added with 5 mM ethylenediaminetetraacetic acid (EDTA) for two times. The filtrated fragments were then digested in RPMI 5% FBS, 1 mg/ml collagenase type II, and 40 μg/ml DNase-I for 40 min. The filtered suspension, containing the cecum lamina propria cells, was centrifuged for 5 min at 1,500 rpm and resuspended in RPMI complete medium. Single-cell suspensions from cecal lamina propria were stained with labeled antibodies diluted in PBS with 2% FBS for 20 min on ice. The following mouse antibodies (mAbs) were used for experiments: APC-conjugated anti-CD11b (1:200) and PE-conjugated anti-GR1 (1:200). Samples were acquired on an LSR Fortessa (BD Biosciences, Franklin Lakes NJ, USA) flow cytometer. Data were analyzed using FlowJo software (TreeStar, Ashland, OR, USA) or FACS Diva software (BD Biosciences, Franklin Lakes NJ, USA).

### Quantification of Fecal Lipocalin-2

The inflammation status of mice was evaluated by measuring the levels of lipocalin-2 (LCN-2) in fecal supernatants via ELISA assay (R&D systems, DuoSet ELISA Mouse Lipocalin-2/NGAL) 24, 48, and 72 h post-infection. Briefly, 0.01 g feces was resuspended in 100 μl in PBS, centrifuged for 10 min at maximum speed, and diluted before performing the ELISA assay, according to manufacturer's instructions.

### Quantification of Fecal IgA by ELISA

ELISA plates were coated (16 h at 4°C) with 75 μl of unlabeled goat anti-murine IgA (Southern Biotech) at 5 μg/ml in PBS, washed four times with PBS 0.025% Tween 20, and saturated with 200 μl of PBS 10% fetal calf serum (FCS) for 2 h at room temperature. Fifty microliters of serial dilutions of stools was incubated for 4 h at room temperature. After four washes in PBS 0.025% Tween 20, 50 μl of alkaline-phosphatase-conjugated goat anti-mouse IgA (Southern Biotech) (1/500 in PBS 10% FCS) were added and plates incubated for 2 h at room temperature. The assay was developed with Sigma 104 phosphatase substrate.

### Histological Evaluation of *C. jejuni* Infections

Ceca from all animals were examined at necropsy, fixed in 10% neutral buffered formalin for at least 48 h prior to embedding in paraffin, and stained with hematoxylin and eosin (H&E). Pathological scores were determined in a blinded manner using a previously described scoring scheme (31). Briefly, each tissue section was assessed for (1) submucosal edema (0, no change; 1, mild; 2, moderate; 3, severe); (2) crypt hyperplasia (0, no change; 1, 1–50%; 2, 51–100%; 3, >100%); (3) goblet cell depletion (0, no change; 1, mild depletion; 2, severe depletion; 3, absence of goblet cells); (4) epithelial integrity [0, no pathological changes detectable; 1, epithelial desquamation (few cells sloughed, surface rippled); 2, erosion of epithelial surface (epithelial surface rippled, damaged); 3, epithelial surface severely disrupted/damaged, large amounts of cell sloughing; 4, ulceration [with an additional score of 1 added for each 25% fraction of tissue in the cross-section affected up to a maximum score of 8 (4 + 4) for a tissue section that had entirely lost its crypt structure due to epithelial cell loss and immune cell infiltration]; (5) mucosal mononuclear cell infiltration (per 400 × magnification field) (0, no change; 1, <20; 2, 20–50; 3, >50 cells/field); (6) submucosal PMN and mononuclear cell infiltration (per 400× magnification field) (1, <5; 2, 21–60; 3, 61–100; 4, >100 cells/field). A maximum score under this scale is 24.

### Statistical Analysis

Statistical analyses were performed using GraphPad Prism v7.04 (GraphPad Software, La Jolla, CA, USA). Mann–Whitney *U* test was used to determine the statistical significance of the results. A *p* < 0.05 was considered significant in all cases.

## Results

### Isolation of FliD-Reactive Monoclonal Antibodies

Different *Campylobacter*'s proteins were evaluated as potential targets to prevent infection on the basis of (i) the presence of surface exposed epitopes, (ii) role in the bacteria pathogenesis, (iii) ability to stimulate an immune response in the context of natural infection, and (iv) level of amino acid sequence conservation within but not limited to the *C. jejuni* species.

The flagellar-capping protein FliD, also known as hook-associated protein 2 (HAP2), is a 70-kDa protein with high sequence conservation across the *C. jejuni* species (26) (Supplemental Figures 1A,B). Based on structures and self-oligomerization mechanisms characterized for other flagellated pathogens (25, 32), *Campylobacter* FliD oligomers form the cap protein complex located at the tip of the flagellum, which controls the distal growth of the filament by regulating the assembly of the flagellin molecules. Due to its functional role in filament elongation, FliD-deficient mutants exhibit defects in bacterial motility (25). The potential of FliD as a promising target is strengthened by the proposed involvement in cell adherence (33) and the immunogenicity in chickens during natural infection. Accordingly, it has been suggested as a vaccine candidate for the prevention *C. jejuni* in broilers (26, 34). FliD was therefore selected as a candidate target antigen for monoclonal antibody (mAb) development.

The frequencies of IgG^+^ and IgA^+^ FliD-reactive memory B cells in 50 tonsillar samples of Swiss origin were evaluated using the antigen-specific memory B cell repertoire analysis (AMBRA) (35) (Supplemental Figure 2). IgG^+^ and IgA^+^ memory B cells from selected tonsils were then immortalized under clonal conditions (28), and culture supernatants were screened using a 384-well plate-based high-throughput platform to identify B-cell clones expressing FliD-reactive antibodies. Using this approach, memory B cell clones producing two human monoclonal antibodies, namely CAA1 and CCG4, were isolated and selected based on their specificity and affinity for recombinant FliD. CAA1 was originally isolated as an IgA1 encoded by V_H_3-48/D2-15/J_H_3 with a 21-amino acid HCDR3 and V_K_1-39/J_K_5, whereas CCG4 was isolated as an IgG3 encoded by V_H_3-9/D1-7/J_H_1 bearing a shorter 11-amino-acid HCDR3 and V_L_3-27/J_L_3.

### Generation and Structural Characterization of FliD-Reactive Recombinant SIgA

Humans present two different IgA subclasses that differ mainly for the length and glycosylation of the hinge region. IgA1 possesses a 13-amino-acid longer hinge than IgA2 containing up to five O-linked glycans at serine and threonine residues. The longer hinge not only confers to IgA1 antibodies higher flexibility and a longer Fab reach but is also linked to the sensitivity to a specific class of bacterial proteases known as IgA1 proteases. Bearing a shorter hinge region lacking proline–serine and/or proline–threonine peptide bonds, IgA2 is instead resistant to these proteases (36). In addition, when compared to IgG, IgM, and IgA1, the IgA2 isotype has the unique characteristic to undergo reverse transcytosis by contacting Dectin-I receptor on the surface of PPs M cells (20). Both the Cα1 region and the glycosylation pattern appear pivotal for the interaction at the basis of this mechanism, which has been suggested as a strategy to boost adaptive immunity against pathogens (37).

The recombinant SIgA1, SIgA2, and dIgA2 versions of the FliD-reactive mAbs were generated by transient transfection of Expi293 cells and were then purified by affinity chromatography. Various physicochemical measurements were performed to monitor key structural attributes of recombinant FliD-reactive polymeric IgAs as summarized in Supplemental Figure 3A. The conformational stability of the mAbs was determined by monitoring their overall tertiary structure as a function of temperature (intrinsic tryptophan fluorescence spectroscopy), and a 6–7°C lower thermal melting temperature (Tm) value was observed for SIgA1 when compared to SIgA2 and dIgA2 (Supplemental Figure 3A). The difference in conformational stability could be due to structural differences in the hinge region between the two subtypes. The relative solubility of the mAbs was evaluated by a PEG precipitation assay, and dIgA2 was found to be relatively more soluble compared to SIgA1 and SIgA2, a result of which suggests that the presence of the secretory component protein may affect mAb solubility at neutral pH. Molecular size analysis by sedimentation velocity analytical ultracentrifugation (SV-AUC) revealed a heterogeneous distribution of species for each mAb (Supplemental Figure 3A). We observed that SIgA2 had a considerably higher relative percent composition of larger molecular weight species than the other two mAbs (as compared to the percent main peak). As expected, dIgA2 had a later elution time when compared to SIgA1 and SIgA2 in size exclusion chromatography (Supplemental Figure 3B). Each of the CAA1 IgA molecules were heavily glycosylated with carbohydrates ranging from ~31–39% of the total mass of each mAb. As expected by sharing the same IgA backbone, the N-linked oligosaccharide structural types were overall very similar in SIgA2 and dIgA2 antibodies (Supplemental Figure 3C). Differently to these antibodies, CAA1 SIgA1 lacked G0F + GN, G1F – GN, GIF + GN and G1F + NANA, while it contained G2F2 and Man4G12 + NANA (Supplemental Figure 3C). Finally, incubation of CAA1 SIgA1 and SIgA2 with recombinant IgAase from *N. gonorrhoeae* confirmed the differences in sensitivity to bacterial IgA proteases of the two isotypes, which have been associated to the length and sequence of the hinge regions (Supplemental Figure 3D).

### *In vitro* Characterization of CAA1 and CCG4 SIgA2

Based on its resistance to bacterial IgA proteases and the ability to undergo reverse transcytosis, the IgA2 scaffold was initially selected over the IgA1 for our studies, and both CAA1 and CCG4 SIgA2 were further characterized *in vitro*. To include a control antibody that was not reactive with the antigen in our experiments of *Campylobacter* infection, we also generated the SIgA2 version of the HGN194 mAb, which targets an epitope on the V3 loop of HIV-1 Env glycoprotein (38).

**Figure 1:**
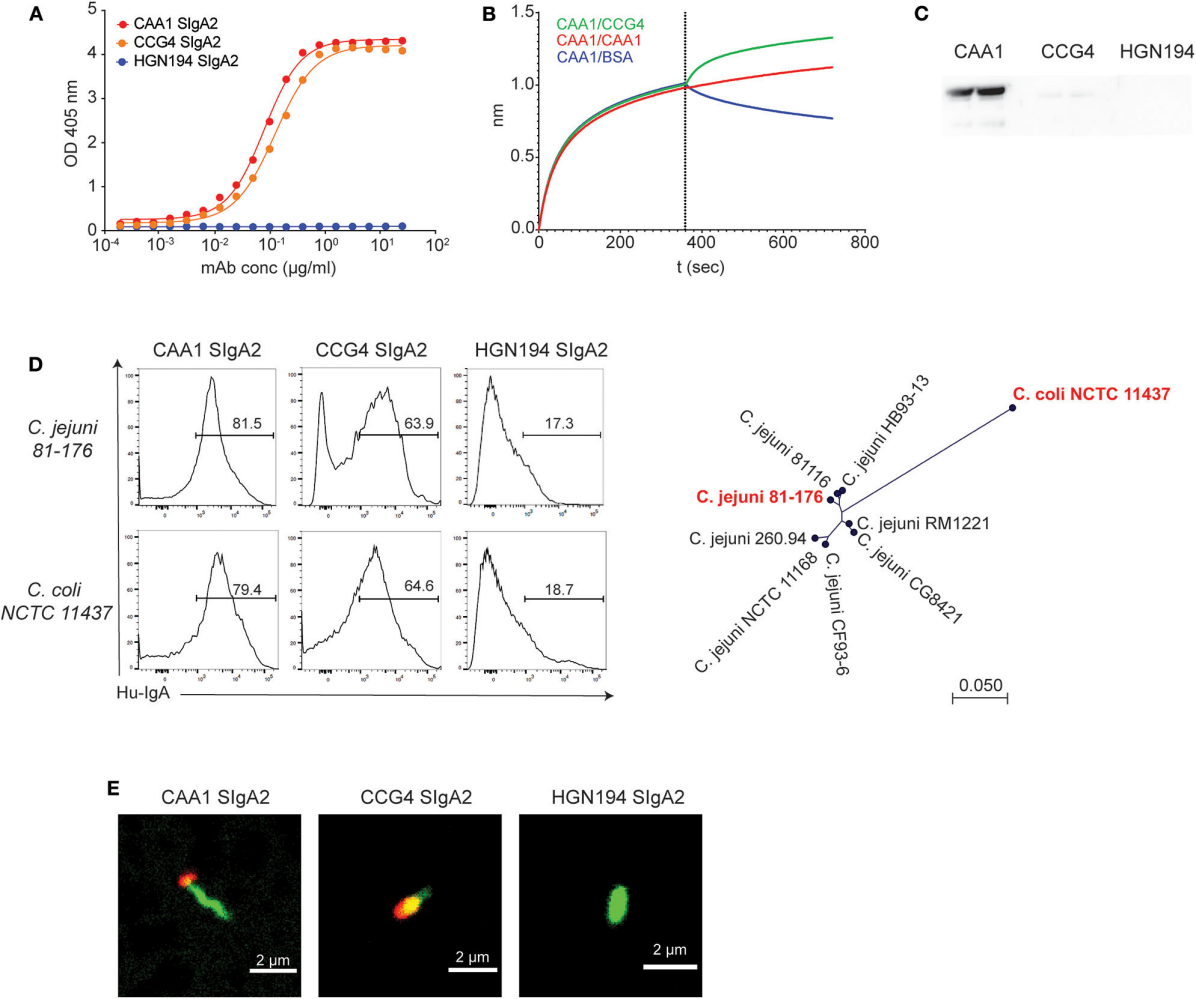
*In vitro* characterization of CAA1 and CCG4 secretory immunoglobulin A (SIgA) binding to FliD. **(A)** Binding of CAA1, CCG4, and HGN194 SIgA to recombinant FliD antigen as measure by ELISA. **(B)** Cross-competition studies performed by biolayer interferometry (BLI). FliD antigen was immobilized on APS sensors and then incubated with CAA1 prior to association with CCG4, CAA1, or phosphate-buffered saline (PBS) with 1% bovine serum albumin (BSA). **(C)** Western blot analysis of CAA1, CCG4, and HGN194 SIgA binding to FliD antigen (70 kDa) under reducing and denaturing conditions. **(D)** Representative histograms of the *in vitro* binding of the indicated mAbs against pure culture of *C. jejuni* 81-176 (ATCC BAA-2151) and *C. coli* NCTC 11437. A phylogenetic tree built using neighbor-Joining method with Jukes–Cantor distance measurement is shown to provide the amino acid distance of FliD between the two historical isolates tested (in red). One representative experiment out of three is represented. **(E)** Binding of CAA1 SIgA to *C. jejuni* as observed in confocal microscopy. Bacteria were stained using Syto BC (green), whereas CAA1 was detected using anti-human IgA AF647 conjugated (red).

CAA1 and CCG4 SIgA2 molecules maintained the original FliD binding activity, displaying similar EC_50_ (CAA1 = 0.08; CCG4 = 0.12 μg/ml) for the flagellar protein (Figure 1A). Epitope binning studies by BLI indicated that the binding of one antibody to the antigen did not prevent the binding of the other mAb (Figure 1B), pointing to the recognition of two distinct epitopes. In addition, CAA1, but not CCG4, recognized FliD by Western blot analysis under denaturing and reducing conditions, confirming the binding to structurally different antigenic determinants (Figure 1C). Accessibility of the two mAbs to these epitopes in the flagellum was assessed by flow cytometry using two FliD-divergent historical isolates *C. jejuni* 81-176 and *C. coli* NCTC 11437. CAA1 and CCG4 stained both pathogens, thus confirming epitope accessibility and breadth (Figure 1D). Consistent with FliD localization, confocal imaging on *C. jejuni* 81-176 confirmed that the binding of the two mAbs occurred at the cell poles (Figure 1E).

### Establishing an Immunocompetent Mouse Model of *Campylobacter* Infection to Test the Prophylactic Potential of FliD-Reactive Antibodies

The murine intestine is highly resistant to *Campylobacter* due to intense competition exerted by the rich gut microbiota and to a certain level of tolerance, which limits inflammation (39–41). To overcome the challenge represented by the resident microbiota, pre-treatment via oral gavage with vancomycin, for which *Campylobacter* species are inherently resistant (42), is largely adopted. Although the pretreatment allows robust bacterial colonization in the cecum, it does not appear to enhance the pathology in adult wild-type mice since minimal signs of inflammation were previously observed (31).

**Figure 2:**
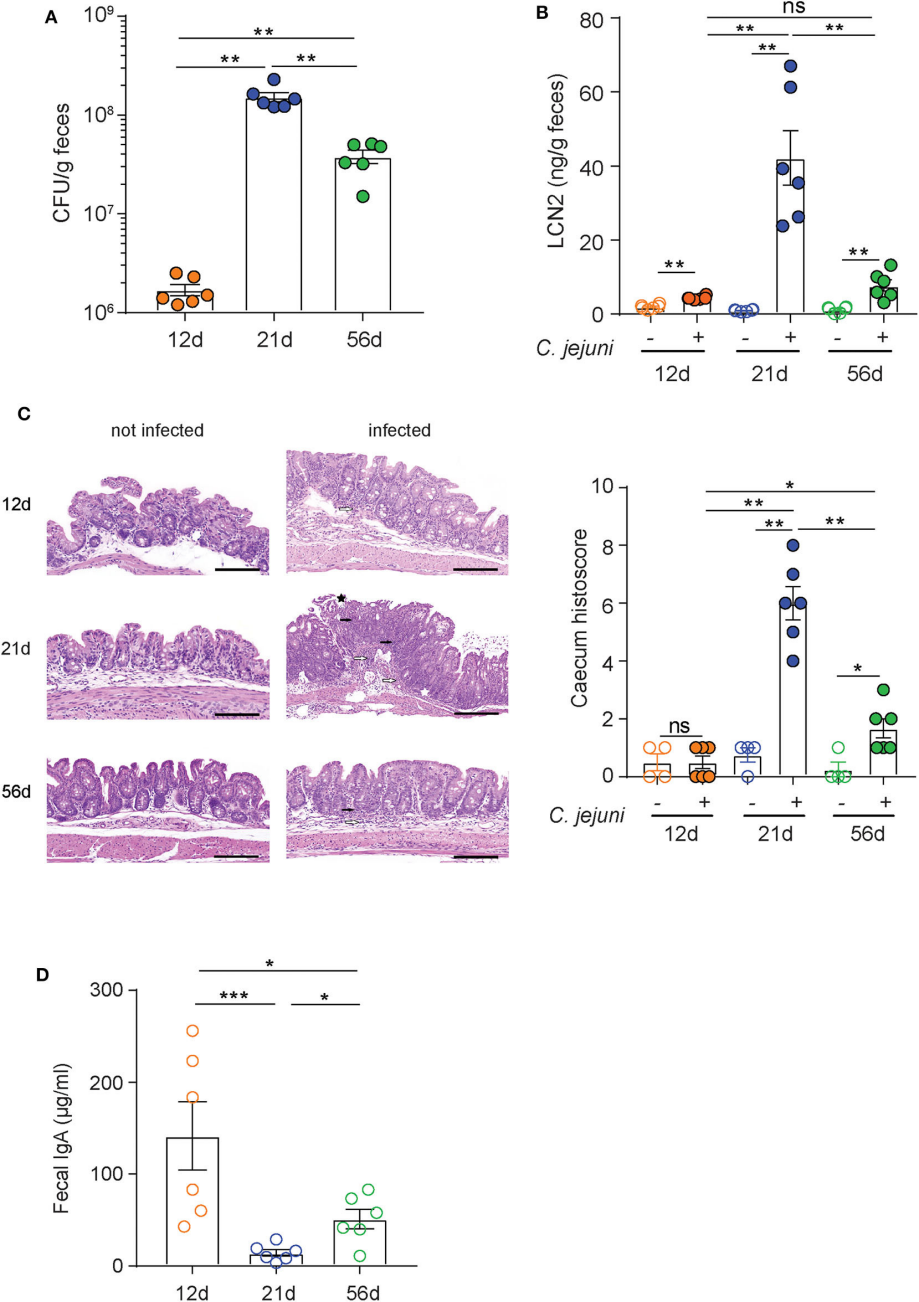
Superior sensitivity of C5BL/6 just weaned mice to *C. jejuni* infection. **(A–C)** C57BL/6 mice at 12, 21, and 56 days of age were orally infected with 10^8^ CFU of *C. jejuni*. **(A)** Bacteria loads (CFU) and **(B)** lipocalin 2 (LCN2) in the stools of infected animals were determined at 6 days postinfection. **(C)** Representative H&E sections of the cecum from infected mice and statistical analysis of histopathological scores at 6 days postinfection. White arrows: submucosal inflammation; white asterisk: crypt hyperplasia with decreased number of goblet cells; black asterisk: epithelial desquamation; black arrows: mucosal inflammation. Scale bar: 200 μm. **(D)** Quantification of fecal immunoglobulin A (IgA) concentrations in C57BL/6 mice at 12, 21, and 56 days of age. Dots represent individual mice and results are shown as ±SEM. **(A–D)** Mann–Whitney test was used. *p < 0.05, **p < 0.01, ***p < 0.001. One representative experiment out of at least two is shown.

Higher susceptibility to *C. jejuni* infection of infant wild-type mice in comparison to adult animals has been previously reported and linked to significant differences in the microbiota composition (43). To set up a model that would allow us to evaluate the prophylactic activity of our mAbs under immunocompetent conditions, we evaluated the sensitivity to *C. jejuni* 81-176 infection of pups (12 days old), weaned (21 days old), and adult (56 days old) C57BL/6 mice. Animals were pre-treated with vancomycin via oral gavage to deplete the murine microbiota before infection with *C. jejuni* 81-176 at 10^8^ CFU/mouse. *Campylobacter* isolation from stools at 6 days post-infection revealed a ~2 log higher increase in shedding from 21-day-old animals than from 12- and 56-day-old mice (Figure 2A). In line with these results, weaned mice displayed significantly higher intestinal inflammation as measured by the levels of lipocalin-2 in the stools and histological score values in the cecum in comparison to pups and adult mice (Figures 2B,C).

Since antibiotic pretreatment should provide comparable ecological niches for infection in the different animals, other factors could be accountable for the different sensitivity to *C. jejuni* infection displayed by the three groups of mice. Analysis of murine IgA in the stools of 12-, 21-, and 56-day-old animals revealed different concentrations among the groups (Figure 2D). Interestingly, weaned mice presented negligible levels of IgA in the stools in comparison to both pups and adult mice. This observation is consistent with the transition between the exogenous supply of maternal antibodies provided along with the milk (12-day-old mice) and the beginning of the endogenous production that is established in adult animals (i.e., 56-day-old mice). Therefore, a likely important factor in the susceptibility of weaned mice to *C. jejuni* infection could be the lower concentration of secretory IgA due to the relative immaturity of intestinal immune system and the depletion of maternal antibodies in these animals. Based on these observations, weaned mice were selected as an immune competent animal model to study the prophylactic activity of FliD-reactive mAbs.

Since off-target binding of CAA1 and CCG4 to the murine microbiota would result in reduced bioavailability and thus activity against pathogens in a prophylactic setting, we evaluated potential cross-reactivity of the recombinant SIgA with the microbiota of weaned mice. Stools from animals orally infected with *C. jejuni, C. coli*, or PBS (mock infected) were collected 24 h post-infection and incubated with the two FliD-reactive mAbs and the control HGN194. CCG4 displayed limited levels of cross-reactivity with the murine microbiota (6.62%), which were comparable with those observed for the control antibody (1.11%) (Supplemental Figure 4A). Conversely, CAA1 was binding the microbiota to a higher extent (22%), suggesting a possible Fab-mediated recognition of antigens in the commensal bacteria that present structures similar to FliD (Supplemental Figure 4A). Nevertheless, for both FliD-reactive mAbs, the percentage of IgA-coated bacteria dramatically increased in the stools of infected animals confirming the prompt recognition of the *C. jejuni* and *C. coli* species (CAA1, 79.2 and 73.9%; CCG4, 70.7 and 55.4%). Interestingly, we also observed an increase in HGN194 binding to the bacteria in the stools of these animals (26.5 and 26.4%) that could be consistent with the innate binding activity of IgA and secretory component against various pathogens (44, 45).

Finally, to examine the conditions for testing the prophylactic efficacy of the mAbs, we evaluated the pharmacokinetics of orally administered SIgA in different gastrointestinal tracts of weaned mice. The *Campylobacter*-irrelevant HGN194 SIgA2, which displayed negligible cross-reactivity with the murine microbiota (Supplemental Figure 4A), was administered as a single oral gavage of 150 μg in PBS (≈15 mg/kg), and its concentration in the different intestinal subcompartments was measured at 2, 4, and 8 h post-administration (Supplemental Figure 4B). HGN194 SIgA concentration in the cecum was maintained almost constant within the first 4 h post-administration and then tended to rapidly decrease by 8 h. The human antibody was not detectable at 12 or 24 h post-administration (data not shown).

### Prophylactic Administration of FliD-Reactive SIgA Accelerates *C. jejuni* Clearance and Reduces Intestinal Inflammation

**Figure 3:**
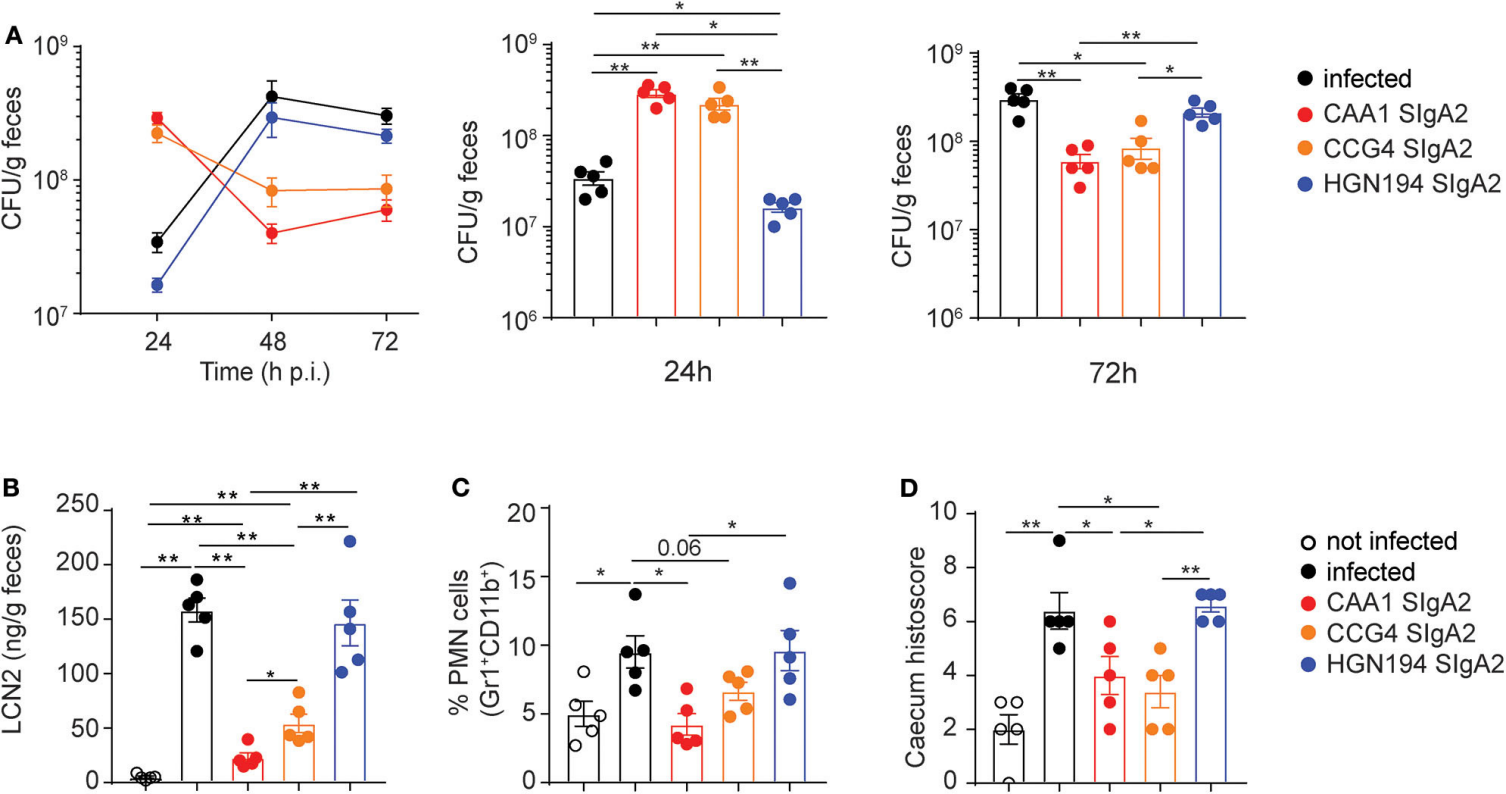
Prophylactic activity of orally administered CAA1 and CCG4 secretory immunoglobulin A (SIgA) against *C. jejuni* infection in just weaned mice. **(A–D)** Two hours prior to infection with 10^8^ CFU of *C. jejuni*, 21-day-old C57BL/6 mice were orally administered by gavage with 200 μg of the indicated mAbs in phosphate-buffered saline (PBS). **(A)** Fecal bacterial load (CFU) at 24, 48, and 72 h post-infection were determined. **(B)** Lipocalin-2 (LCN2) levels in the stools, **(C)** statistical analysis of polymorphonucleated (PMN) cell infiltrates gated as Gr1^+^CD11b^+^, and **(D)** histopathological score in the caecum were determined at 72 h post-infection in the different treatment conditions. Dots represent individual mice, and results are shown as ±SEM. **(A–D)** Mann–Whitney test was used. *p < 0.05, **p < 0.01. One representative experiment out of at least two is shown.

We next evaluated the prophylactic activity of orally administered FliD-reactive recombinant SIgA2 in immunocompetent-weaned mice infected with *C. jejuni* 81-176. As such, vancomycin pretreated animals were administered with a single oral gavage of 200 μg/mouse of CAA1, CCG4, HGN194 SIgA2, or PBS 2 h before oral infection with 10^8^ CFU/mouse of *C. jejuni* 81-176. Treated animals and the corresponding control groups were monitored for 72 h, during which the severity of infection and degree of inflammation were recorded. Of note, analysis of the stools from treated mice revealed a trend characterized by higher *Campylobacter* shedding at 24 h post-infection followed by a significant decrease over time. Conversely, untreated and HGN194-treated groups presented lower shedding at early time points followed by a consistent CFU increase at 48 h post-infection (Figure 3A).

These results suggest that that CAA1 and CCG4 could prevent or reduce the ability of the pathogen to adhere to the surface of the mucosal epithelium, thus facilitating the clearance of bacteria via peristalsis or mucociliary activity at early stages post-infection. This hypothesis is further supported by the significant lower levels of lipocalin-2, a marker of intestinal inflammation linked to epithelial damage and neutrophil infiltration, recorded at 72 h post-infection in the stools of CAA1- and CCG4-treated animals in comparison to the control groups (infected/non-treated and infected/HGN194-treated groups) (Figure 3B). In addition, animals treated with a single administration of FliD-reactive mAbs presented levels of PMN cells infiltration and histological score values in the cecum significantly lower than the HGN194-treated group (Figures 3C,D), which indicates that the activity exerted by CAA1 and CCG4 is largely Fab mediated.

Similar findings were observed in animals administered 2 h before infection with higher or lower *Campylobacter* infection doses (10^7^ or 10^9^ CFU/mouse). Indeed, in both cases CAA1-administered animals presented significantly higher bacterial clearance than the untreated group (Supplemental Figures 5A,B) and levels of intestinal inflammation similar to those of noninfected mice (Supplemental Figures 5C,D). Furthermore, no significant differences were observed in the reduction in bacterial shedding, PMN infiltration in the cecum lamina propria, and lipocalin-2 levels in the stools when CAA1 SIgA2 was provided 2 h before infection or when it was premixed with *C. jejuni* 81-176 before oral administration to the animals (Supplemental Figures 5E–G).

Taken together, these results indicate that prophylactic administration of FliD-specific CAA1 and CCG4 SIgA2 increase protection from *Campylobacter* infection by accelerating bacterial clearance at early stages after infection, thus significantly reducing inflammation.

### IgA Isotype Switch Does Not Affect CAA1 Prophylactic Activity

Since longer Fab reach, higher flexibility, and different glycosylation may affect the cross-linking ability and/or persistence of the polymeric Ig in the intestine, we next investigated whether the two IgA isotypes may exert different prophylactic activities in our mouse model of *Campylobacter* infection.

**Figure 4:**
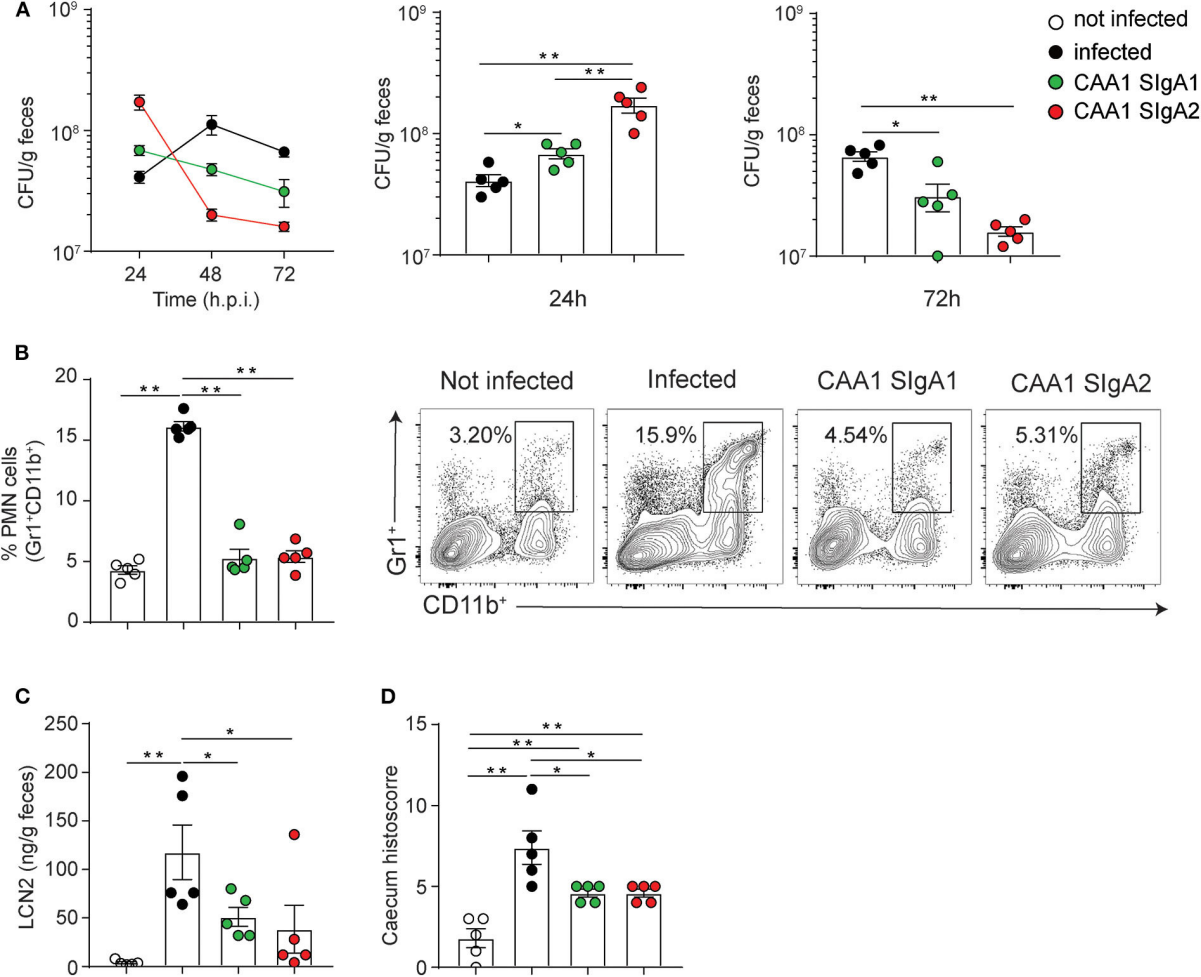
CAA1 SIgA1 and SIgA2 display similar prophylactic activity against *C. jejuni* infection. **(A–D)** Two hours prior to infection with 10^8^ CFU of *C. jejuni*, 21-day-old C57BL/6 mice were orally administered via gavage with 200 μg of CAA1 as SIgA1 or SIgA2. **(A)** Quantification of the bacterial load (CFU) in the stools of the animals at 24, 48, and 72 h post-infection. **(B)** Representative dot plot and relative quantification of polymorphonucleated cells infiltrated in the caecum gated as Gr1^+^CD11b^+^. **(C)** Quantification of lipocalin-2 (LCN2) in the stools, and **(D)** statistical analysis of histopathological score in the cecum at 72 h post-infection in the different treatment conditions. Dots represent individual mice, and results are shown as ±SEM. **(A–D)** Mann–Whitney test was used. *p < 0.05, **p < 0.01. One representative experiment out of at least two is shown.

CAA1 SIgA1 and SIgA2 displayed comparable binding to FliD (Supplemental Figure 6A), and no significant differences in reactivity to *Campylobacter* species *in vitro* or with murine microbiota *ex vivo* were observed between the two formats (Supplemental Figures 6B,C). The prophylactic activity of the two isotypes was then tested in the murine model of *Campylobacter* infection. In line with our previous findings, animals administered with the FliD-reactive mAbs displayed higher *Campylobacter* shedding at early time postinfection followed by decrease over time, while infected non-treated animals produced an opposite trend (Figure 4A).

Although CAA1 SIgA2 accelerated shedding faster than SIgA1 as confirmed also by the percentage of human IgA-coated bacteria in the stools (Supplemental Figure 6D), both subclasses were equally capable to limit inflammation in infected animals as shown by the levels of lipocalin-2 in the stools, PMN infiltration, and the corresponding histological score in the cecum at 72 h postinfection (Figures 4B–D).

Overall, our results indicate that differences in flexibility and glycosylation associated to the two human IgA subclasses do not have an impact in the prophylactic activity exerted by CAA1 in the *in vivo* model.

### SIgA to IgG Conversion Is Detrimental for CAA1 and CCG4 Prophylactic Activity

Although SIgA are the most abundant antibodies expressed in association with the intestinal mucosa and the first line of defense against enteric pathogens, they are characterized by a complex structure, and their development as drugs might result challenging in comparison to IgG-based monoclonal antibodies. Because the activity of the *Campylobacter*-reactive mAbs was previously shown to be dependent on the specificity for FliD, we generated CAA1, CCG4, and the *Campylobacter*-irrelevant antibody HGN194 as human IgG1 and evaluated their prophylactic activity in comparison to their corresponding SIgA2 counterparts.

Since glycosylation might affect the ability of mAbs to interact with mucin on the mucosal surface, the localization and persistence of HGN194 IgG1 in the murine intestinal tract were appraised by administering the antibody with a single oral gavage to weaned mice and then by measuring the its concentration in the different intestinal subcompartments after 2, 4, and 8 h (Supplemental Figure 4C). As in the case of HGN194 SIgA2, at every time point, the highest concentration of the IgG1 was detected in the cecum; however, in this intestinal subcompartment, the IgG1 concentration tended to decrease faster than for SIgA2, with a significant reduction already at 4 h post-administration (Supplemental Figures 4B,C).

**Figure 5:**
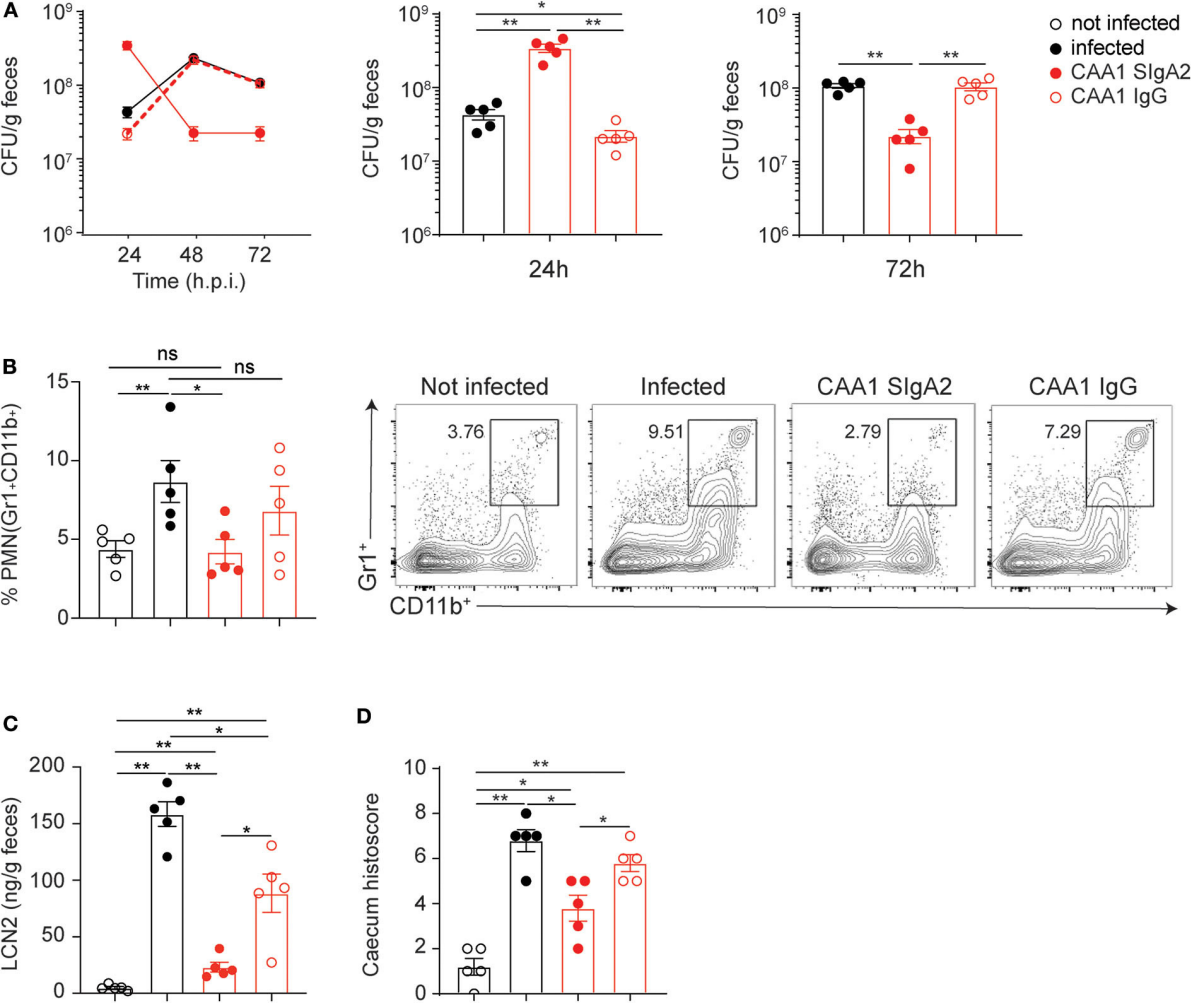
Conversion to immunoglobulin G (IgG) reduces CAA1 prophylactic activity against *C. jejuni* infection. **(A–D)** Two hours prior to infection with 10^8^ CFU of *C. jejuni*, 21-day-old C57BL/6 mice were orally administered via gavage with 200 μg of CAA1 as SIgA2 or IgG1. **(A)** Quantification of the bacterial load (CFU) in the stools of the animals at 24, 48, and 72 h post-infection. **(B)** Representative dot plot and relative quantification of polymorphonucleated cells infiltrated in the cecum gated as Gr1^+^CD11b^+^. **(C)** Quantification of lipocalin-2 (LCN2) in the stools, and **(D)** statistical analysis of histopathological score in the cecum at 72 h post-infection in the different treatment conditions. Dots represent individual mice, and results are shown as ±SEM. **(A–D)** Mann–Whitney test was used. *p < 0.05, **p < 0.01. One representative experiment out of at least two is shown.

Next, we evaluated the prophylactic activity of the FliD-reactive mAbs CAA1 and CCG4 as IgG1 or SIgA2 both administered to weaned mice 2 h before infection with *C. jejuni* 81-176. Interestingly, while animals treated with SIgA2 antibodies displayed the previously observed pattern characterized by higher shedding at 24 h postinfection followed by a significant decrease at 48 and 72 h, the groups treated with the IgG version of the same antibodies revealed trends similar to the non-treated groups (Figure 5A and Supplemental Figure 7A). The importance of the SIgA format for the *in vivo* efficacy was further confirmed by the lower ability of CAA1 and CCG4 IgG to limit inflammation in comparison to their polymeric counterparts, as shown by PMN cells infiltration in the cecum and lipocalin-2 levels in the stools of the infected animals at 72 h post-infection (Figures 5B,C and Supplemental Figures 7B,C). Overall, no significant differences in the histological scores were observed between the mice treated with the FliD-reactive IgG antibodies and the non-treated animals (Figure 5D and Supplemental Figure 7D). Conversely, the SIgA2 versions of the antibodies were able to replicate the beneficial effect previously observed, maintaining the histological score in the cecum to values significantly lower than both non-treated and IgG-treated mice (Figure 5D and Supplemental Figure 7D).

Taken together, these data indicate that the CAA1 and CCG4 IgG have limited prophylactic activity when orally administered prior to *Campylobacter* infection. The reduced activity of CAA1 and CCG4 IgG might be dependent on both the lower stability and persistence in the gastrointestinal tract and/or on the decreased “bonus effect of multivalency” at the basis of the cross-linking activity associated with the polymeric format.

## Discussion

The aim of this study was to evaluate the ability of orally administered recombinant SIgA, derived from selected FliD-reactive monoclonal antibodies, to prevent infections by *Campylobacter* species frequently associated with severe neonatal gastroenteritis.

Despite its pivotal role in bacteria motility, epithelial cells adherence (25, 33), and the high level of conservation among *C. jejuni* (Supplemental Figure 1), FliD has never been assessed to date as a potential target for therapeutic mAbs. Consistent with the immunogenicity previously reported for this antigen (26, 34), our results indicate that FliD-reactive mAbs can be isolated from the human memory B cell repertoire. Further development of these antibodies, namely, CAA1 and CCG4, resulted in recombinant SIgA that preserved the ability to bind FliD on the *Campylobacter*'s flagellum of both *C. jejuni* and *C. coli* species (Figure 1D).

Genetically manipulated animals characterized by an exacerbated inflammatory response to bacteria, such as SIGIRR or IL10^−^/^−^, have been proposed as models to study *Campylobacter* pathogenesis (31, 46). These mutations, however, dramatically alter the murine immune system to an extent that even the presence of commensal microbes can potentially result in spontaneous enterocolitis (47). The sensitivity of wild-type mice to *Campylobacter* infection has consistently been linked to age-dependent variations in the composition of the resident microbiota (43). Of note, our findings indicate that different levels of IgA in the murine intestine also play a role in *Campylobacter* pathogenesis in mice. In particular, we observed that weaned mice represent the most permissive wild-type age group and that this is likely due to the transition between the exogenous IgA supply provided by the maternal milk and IgA endogenous production (Figure 2D). Moreover, Giallourou et al. described a weaned mouse model of *C. jejuni* that can recapitulate enteropathy and diarrhea (48). In the current study, we established a weaned mouse model of *Campylobacter* infection to evaluate the prophylactic activity of the mAbs based on bacterial shedding and inflammatory biomarkers (Figures 2A–C). In this animal model, we documented a consistent effect following a single prophylactic administration of FliD-reactive SIgA 2 h before *C. jejuni* infection, which is characterized by significantly higher *Campylobacter* shedding at the early stage of infection followed by a rapid decline at later time points (Figure 3A). Our hypothesis is that by binding FliD, the SIgA can limit bacteria motility and ability to adhere to the surface of the mucosal epithelium, thus accelerating their clearance via peristalsis. This idea is further strengthened by the fact that animals administered with FliD-reactive mAbs display considerably lower levels of inflammatory biomarkers in the cecum and stools than the control groups, which present an opposite shedding trend. FliD-reactive mAbs efficacy relies on the specific recognition of the bacterial surface antigen, as an irrelevant antibody in the same format does not produce similar beneficial effects (Figures 3A–D). Further studies will elucidate whether these antibodies may act primarily by limiting bacteria motility (cross-linkage), by limiting attachment to cell receptors (steric hindrance), by limiting secretion via the T3SS of proteins that contribute to host cell invasion (e.g., CiaB) or via a combination of all these activities. Interestingly, similar prophylactic activity was observed when CAA1 was premixed with *C. jejuni* or administered 2 h before infection, thus confirming SIgA stability during the passage through the small intestine and their persistence in the cecum (Supplemental Figures 5E–G).

Despite the fact that SIgA2 showed a slightly higher ability to accelerate bacterial shedding at early stage of infection in comparison to the SIgA1 counterpart (Figure 4A), differences in length and glycosylation of the hinge region between the two human IgA isotypes do not appear to significantly undermine the ability of the polymeric immunoglobulins to prevent inflammation (Figures 4B–D). However, IgA2 might represent the most promising backbone for development of orally deliverable mAbs due to resistance to IgA proteases expressed by different pathogens (Supplemental Figure 3D) and to the unique ability to undergo reverse transcytosis (20), which in turn could contribute to boost a potential vaccinal effect (37).

The prophylactic activity of FliD-reactive CAA1 and CCG4 was significantly decreased when these mAbs were administered as IgG1. Indeed, in the IgG1 format, the mAbs were neither able to boost *Campylobacter* shedding at early time postinfection nor able to limit inflammation to an extent comparable with their corresponding SIgA (Figure 5 and Supplemental Figure 7). The superior efficacy of CAA1 and CCG4 as SIgA might be related to higher resistance to proteases in the small intestine (30, 49), higher localization and better persistence in the cecum (17, 18), and/or to the mechanisms of action (i.e., cross-linking) associated with the polymeric immunoglobulin structure (14, 19).

In summary, our results indicate that the flagellar-capping protein FliD represents a promising target for the development of *Campylobacter*-reactive mAbs capable to exert a prophylactic activity dependent on both their affinity to the antigen and on features strictly related to the SIgA format. Although improvement in water sanitation and healthcare conditions are pivotal to reduce *Campylobacter*'s impact in infants in middle- and low-income countries, these findings suggest that the development of orally deliverable recombinant SIgA targeting FliD might represent a novel strategy to prevent or mitigate both disease severity and the development of post-infection syndromes.

## Data Availability Statement

The datasets generated for this study are available on request to the corresponding author.

## Ethics Statement

The animal study was reviewed and approved by Swiss Federal Veterinary Office guidelines and the Cantonal Veterinary Office (TI-09-2019).

## Author Contributions

LP, FG, DC, and MP conceived the study. LP and MP initiated the study design. SJ, GL, DP, FSt, RP, and FB helped with implementation. DM and FSe provided expertise in microscopy and immunohistochemistry, respectively. YH, SB, JX, OK, SBJ, and DV performed the physiochemical characterization of the recombinant mAbs. LP, FG, DC, and MP outlined and wrote the manuscript, whereas OK, SBJ, and DV contributed to its finalization. All authors approved the final manuscript.

## Conflict of Interest

SJ, GL, DP, FB, DC, and MP are employees of Vir Biotechnology Inc. and may hold shares in Vir Biotechnology Inc. The remaining authors declare that the research was conducted in the absence of any commercial or financial relationships that could be construed as a potential conflict of interest.
