# Supplementary material for: Prophylactic Activity of Orally Administered FliD-Reactive Monoclonal SIgA Against Campylobacter Infection
Source: Front Immunol. 2020 Jun 9;11:1011. doi: 10.3389/fimmu.2020.01011 (PMC7296071; doi:10.3389/fimmu.2020.01011)
Supplement: Supplementary file 1 [file Data_Sheet_1.pdf]

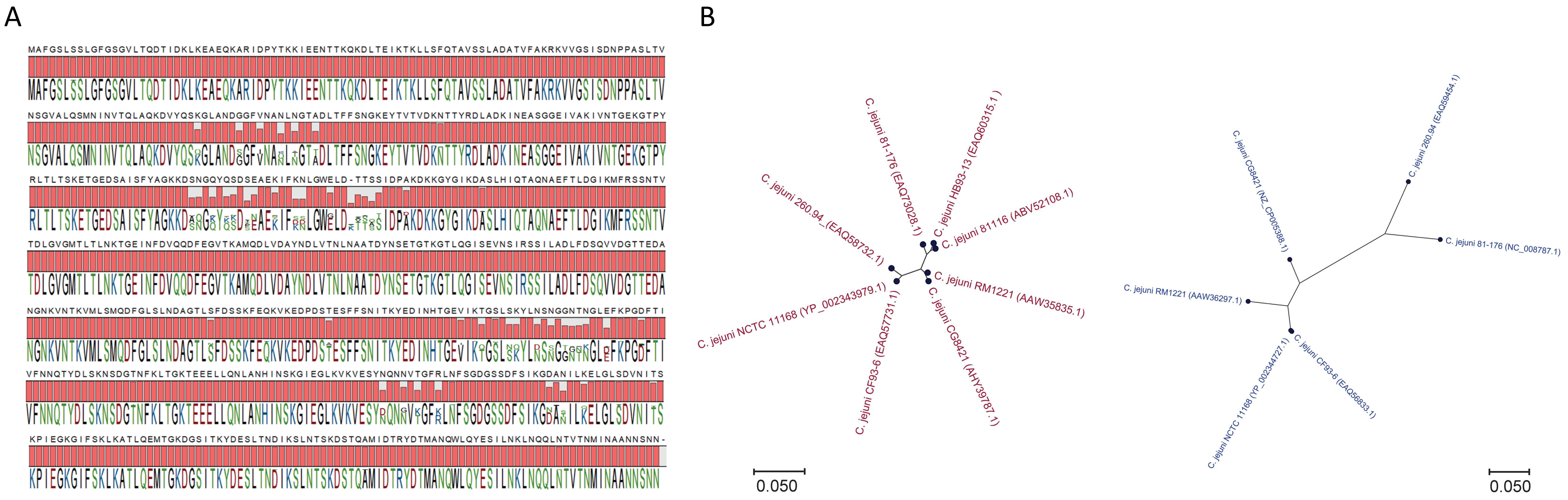

Supplemental Figure 1. Analysis of FliD amino acid sequence conservation.
(A) FliD amino acid sequences from 35 C. jejuni isolates were retrieved from GenBank and analyzed using CLC Main Workbench software (Qiagen). The red bars and the sequence logo underneath represent the percentage of conservation of each amino acid and the potential substitutions at the specific site. The consensus sequence for FliD is reported on top of each line. **(B)** Phylogenetic amino acid distance of FliD from different C. jejuni isolates previously described in literature (50-52) (left panel). Phylogenetic distance of flagellin subunit A (FlaA) for the same isolates is presented for comparison (right panel). The trees were generated using Neighbor-Joining method with Jukes-Cantor distance measurement.
