# Supplementary material for: Prophylactic Activity of Orally Administered FliD-Reactive Monoclonal SIgA Against Campylobacter Infection
Source: Front Immunol. 2020 Jun 9;11:1011. doi: 10.3389/fimmu.2020.01011 (PMC7296071; doi:10.3389/fimmu.2020.01011)
Supplement: Supplementary file 2 [file Data_Sheet_2.PDF]

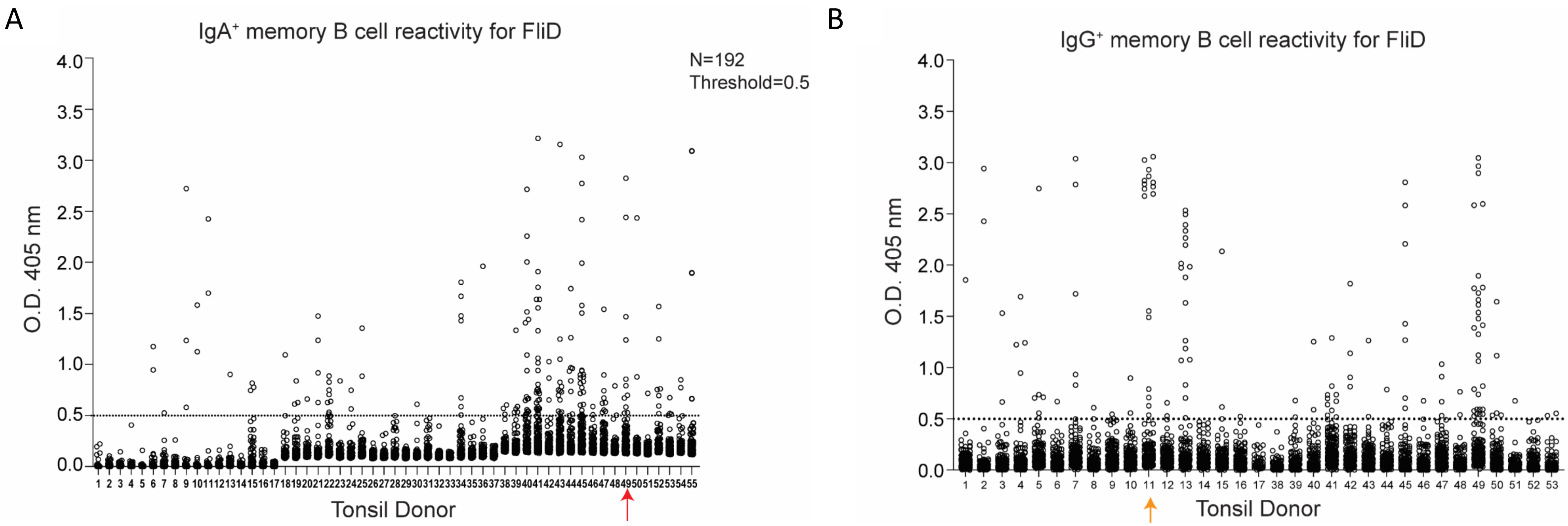

Supplemental Figure 2. Frequency of FliD-reactive IgA+ and IgG+ memory B cell from different tonsillar samples. Analysis of the reactivity against Campylobacter FliD antigen of the IgA^{+} **(A)** and IgG^{+} **(B)** memory B cell repertoire of different tonsillar samples. Arrows indicate the tonsillar donor from which CAA1 (red) and CCG4 (orange) were isolated.
