# Supplementary material for: Prophylactic Activity of Orally Administered FliD-Reactive Monoclonal SIgA Against Campylobacter Infection
Source: Front Immunol. 2020 Jun 9;11:1011. doi: 10.3389/fimmu.2020.01011 (PMC7296071; doi:10.3389/fimmu.2020.01011)
Supplement: Supplementary file 3 [file Data_Sheet_3.pdf]

A

Structural Attribute	Analytical Method	CAA1 mAb SIgA1	SIgA2	dIgA2
Conformational Stability	Tm value by intrinsic fluorescence spectroscopy, (°C) (n=3 ± SD)	72.0 ± 0.4	79.0 ± 0.5	78.5 ± 0.7
Relative Solubility	PEG precipitation assay, (% PEG midpoint) (n=2 ± data range)	5.2 ± 0.1	5.1 ± 0.1	6.2 ± 0.1
Molecular Size	% Main species by SV-AUC, (%) (~11 Svedbergs) (n=2 ± data range)	27 ± 1	7 ± 1	26 ± 2
	Higher molecular weight species by SV-AUC, (%) (>12 Svedbergs) (n=2 ± data range)	67 ± 2	91 ± 4	64 ± 2
	Lower molecular weight species SV-AUC, (%) (<8 Svedbergs) (n=2 ± data range)	6 ± 1	2 ± 1	10 ± 1
Glycosylation	Total Carbohydrate Analysis, (%) (n=2 ± data range)	38.9 ± 0.2	30.7 ± 0.1	32.0 ± 0.1
	N-Glycan Oligosaccharide Analysis, (# of N-glycans) (n=3)	20	21	21

B

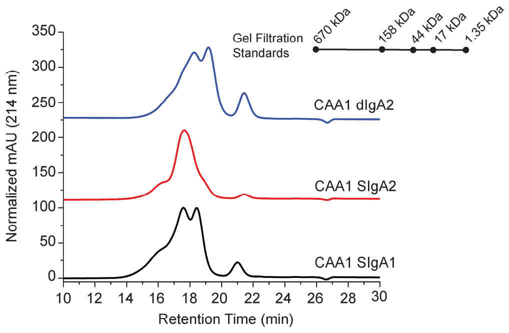

C

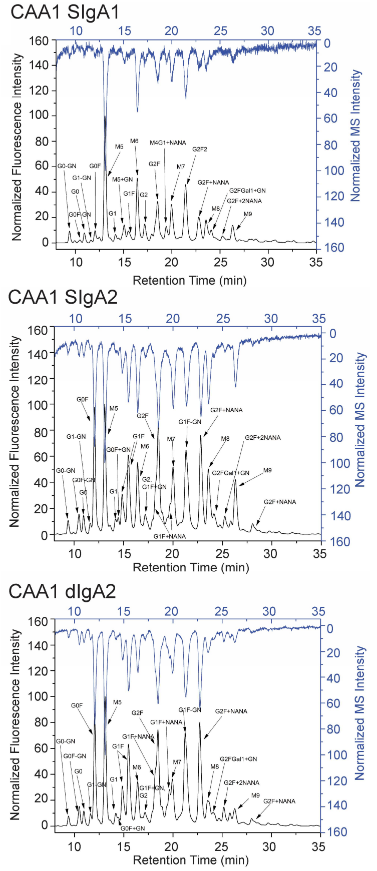

Common Name	Oxford Notation	CFG	Percentage (%) in CAA1 SIgA1	Percentage (%) in CAA1 SIgA2	Percentage (%) in CAA1 dIgA2
G0-GN	A1		1.4	0.8	0.6
G0F-GN	FA1		0.5	1.4	1.4
G0	A2		1.3	1.2	1.1
G1-GN	A1G1		0.4	0.6	0.8
G0F	FA2		1.9	8.5	8.1
G0F+GN	FA3		-	1.0	0.6
G1	A2G1		1.3	1.0	1.0
G1F	FA2G1		2.9	10.1	12.0
G1F-GN	FA1G1		-	10.7	12.9
G1+GN	FA3G1		-	2.2	1.7
G2	A2G2		3.5		
G2F	FA2G2		10.0	9.6	8.4
G2F2	FA2F1G2		12.7	-	-
G2FGal1+GN	FA2BG2Ga1		2.1	2.2	1.8
G1F+NANA	FA2G1S1		-	4.7	8.3
G2F+NANA	FA2G2S1		6.8	10.7	13.0
G2F+2NANA	FA2G2S2		1.7	1.9	2.1
Man4G1+NANA	M4A1G1S1		3.2	-	-
Man5	M5		19.5	9.0	10.4
Man5+GN	M5A1		3.5	-	-
Man6	M6		10.9	6.4	4.9
Man7	M7		7.6	6.0	4.8
Man8	M8		5.0	6.5	3.8
Man9	M9		3.8	5.5	2.1

D

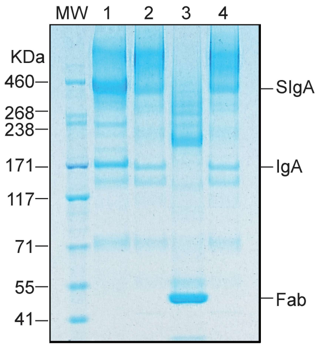

Supplemental Figure 3. Structural characterization of recombinant SIgA.

(A) Summary of the biophysical characteristics of CAA1 mAb in the SIgA1, SIgA2, and dIgA2 formats. Various measurements included conformational stability by intrinsic fluorescence spectroscopy vs temperature, relative solubility by PEG precipitation assay, size analysis by SV-AUC and glycosylation by total carbohydrate analysis and N-glycan oligosaccharide analysis. Sample measurements were performed in PBS, pH 7.2 with mean (n=2 except Tm values and N-linked glycan analysis n=3) ± range. **(B)** Representative size exclusion chromatograms. Absorbance traces (214 nm) of the various CAA1 mAbs as a function of retention time of SEC column are shown. Gel filtration standards are shown for comparison. **(C)** Representative N-Glycosylation analysis of CAA1 SIgA1, SIgA2, and dIgA2 mAbs. Representative profiles of Fluor-MS N-linked glycans that were released from mAbs as measured by fluorescence and mass spectrometry as indicated. All data are presented as mean ± SD; n = 3. A summary of the percentage of the N-glycans for each mAb is indicated in the table. The percentages are the mean of three measurements and the standard deviation of each measurement was ≤1.5%. **(D)** Denaturing non-reducing gel of CAA1 SIgA1 and SIgA2 incubated over-night (18 hours) with PBS or with rIgA protease (IgAse Pro-Pro-Y-Pro) from *Neisseria gonorrhoeae*. (1: SIgA1 + PBS; 2: SIgA2 + PBS; 3: SIgA1+Igase; 4: SIgA2+Igase).
