# Supplementary material for: Prophylactic Activity of Orally Administered FliD-Reactive Monoclonal SIgA Against Campylobacter Infection
Source: Front Immunol. 2020 Jun 9;11:1011. doi: 10.3389/fimmu.2020.01011 (PMC7296071; doi:10.3389/fimmu.2020.01011)
Supplement: Supplementary file 4 [file Data_Sheet_4.PDF]

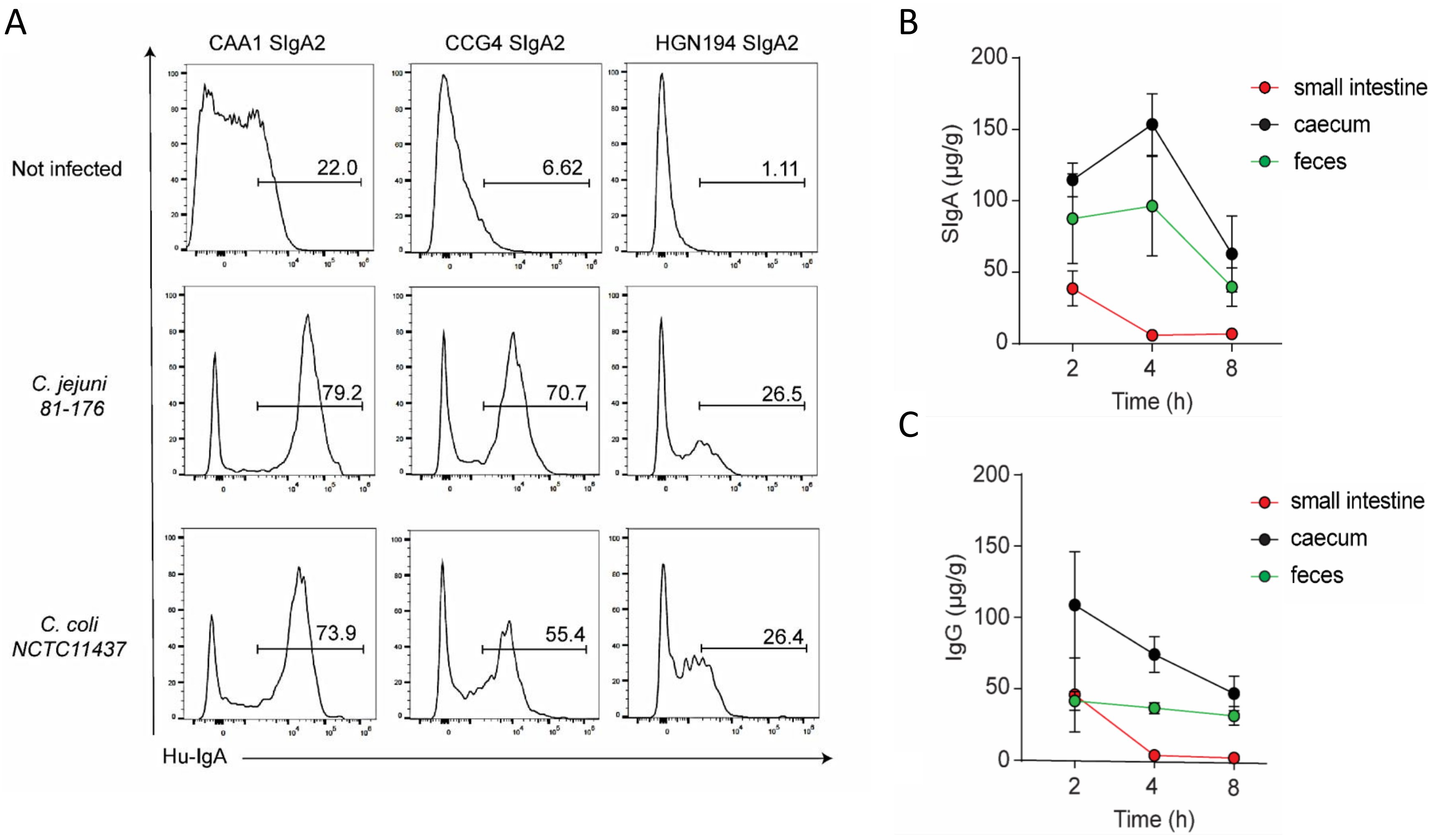

Supplemental Figure 4. Analysis of FliD-reactive mAbs cross-reactivity with the murine microbiota and persistence in caecum of C57BL/6 just weaned mice.

(A) Representative histograms of the specific binding of the indicated mAbs against fecal microbiota of mice mock infected or infected with *C. jejuni* or *C. coli*. One representative experiment out of three is shown. **(B-C)** Pharmacokinetics evaluation by ELISA of HGN194 (B) SIgA and (C) IgG antibody at the indicated time points in the different mouse intestinal sub-compartments. One representative experiment out of at two is shown.
