# Supplementary material for: Prophylactic Activity of Orally Administered FliD-Reactive Monoclonal SIgA Against Campylobacter Infection
Source: Front Immunol. 2020 Jun 9;11:1011. doi: 10.3389/fimmu.2020.01011 (PMC7296071; doi:10.3389/fimmu.2020.01011)
Supplement: Supplementary file 5 [file Data_Sheet_5.pdf]

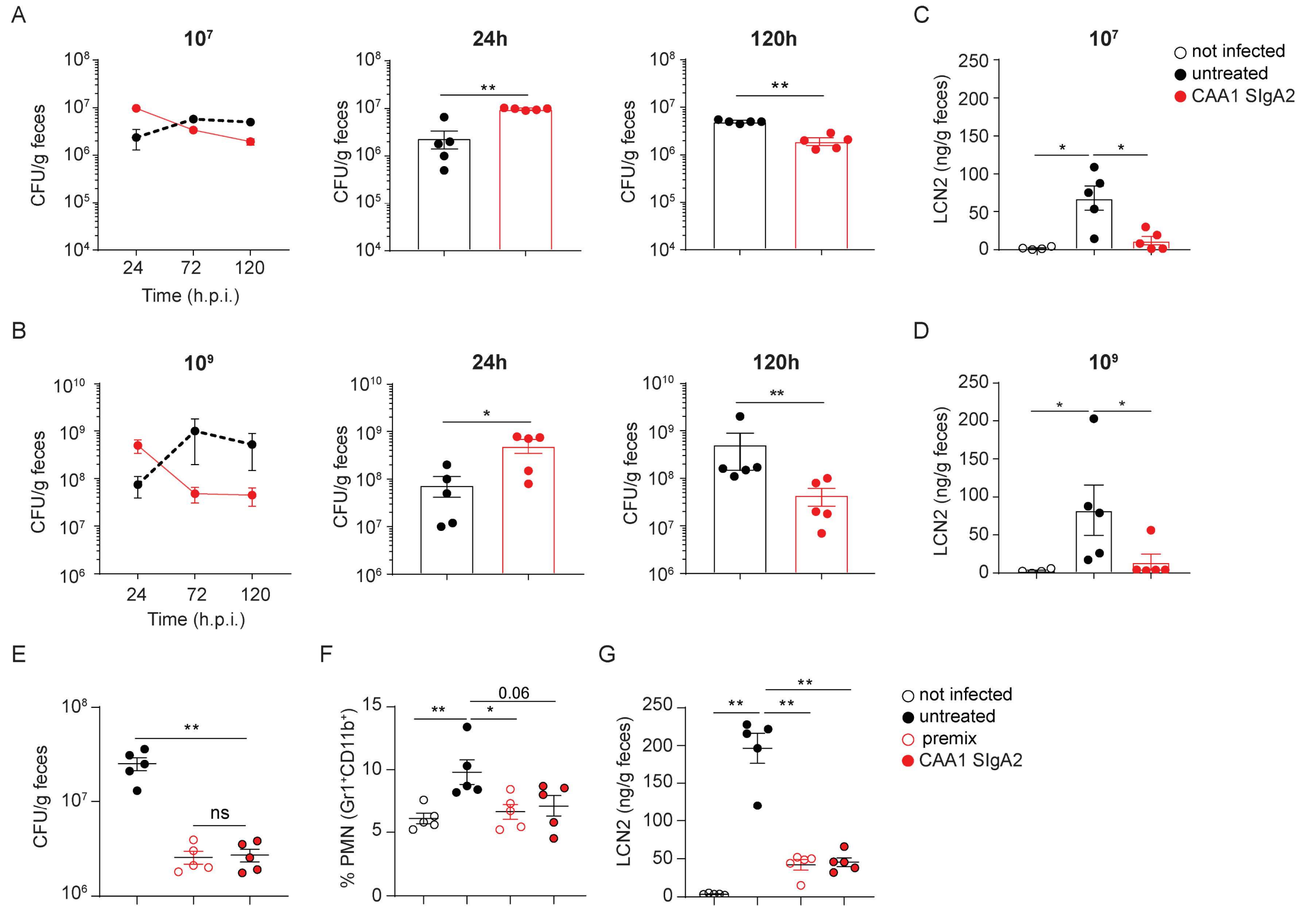

Supplemental Figure 5. Prophylactic activity of CAA1 SIgA at different *C. jejuni* infection doses.

(A-B) Quantification of the fecal bacterial load (CFU) in 21-day-old C57BL/6 mice administered via gavage with 200 µg of CAA1 SIgA2 as measured at 24, 72 and 120h post-infection with 10^7 or 10^9 CFU of *C. jejuni*. **(C-D)** Quantification of fecal lipocalin-2 (LCN2) in 21-day-old C57BL/6 mice administered via gavage with 200 µg of CAA1 SIgA2 as measured at 120h post-infection with 10^7 or 10^9 CFU of *C. jejuni*. **(E-G)** The prophylactic activity exerted by oral administration of CAA1 SIgA2 two hours before infection was compared with the one provided by pre-mixing of the mAb with *C. jejuni 81-176*. Quantification of the bacterial shedding **(E)**, polymorphonucleated cells (PMN) infiltration in the caecum, gated as Gr1+CD11b+ **(F)**, and lipocalin-2 (LCN2) in the stools **(G)** are reported. Dots represent individual mice and results are shown as ± SEM. Mann-Whitney test **(A–G)** was used. $*p < 0.05$, $**p < 0.01$. One representative experiment out of at least two is shown.
