# Supplementary material for: Prophylactic Activity of Orally Administered FliD-Reactive Monoclonal SIgA Against Campylobacter Infection
Source: Front Immunol. 2020 Jun 9;11:1011. doi: 10.3389/fimmu.2020.01011 (PMC7296071; doi:10.3389/fimmu.2020.01011)
Supplement: Supplementary file 6 [file Data_Sheet_6.pdf]

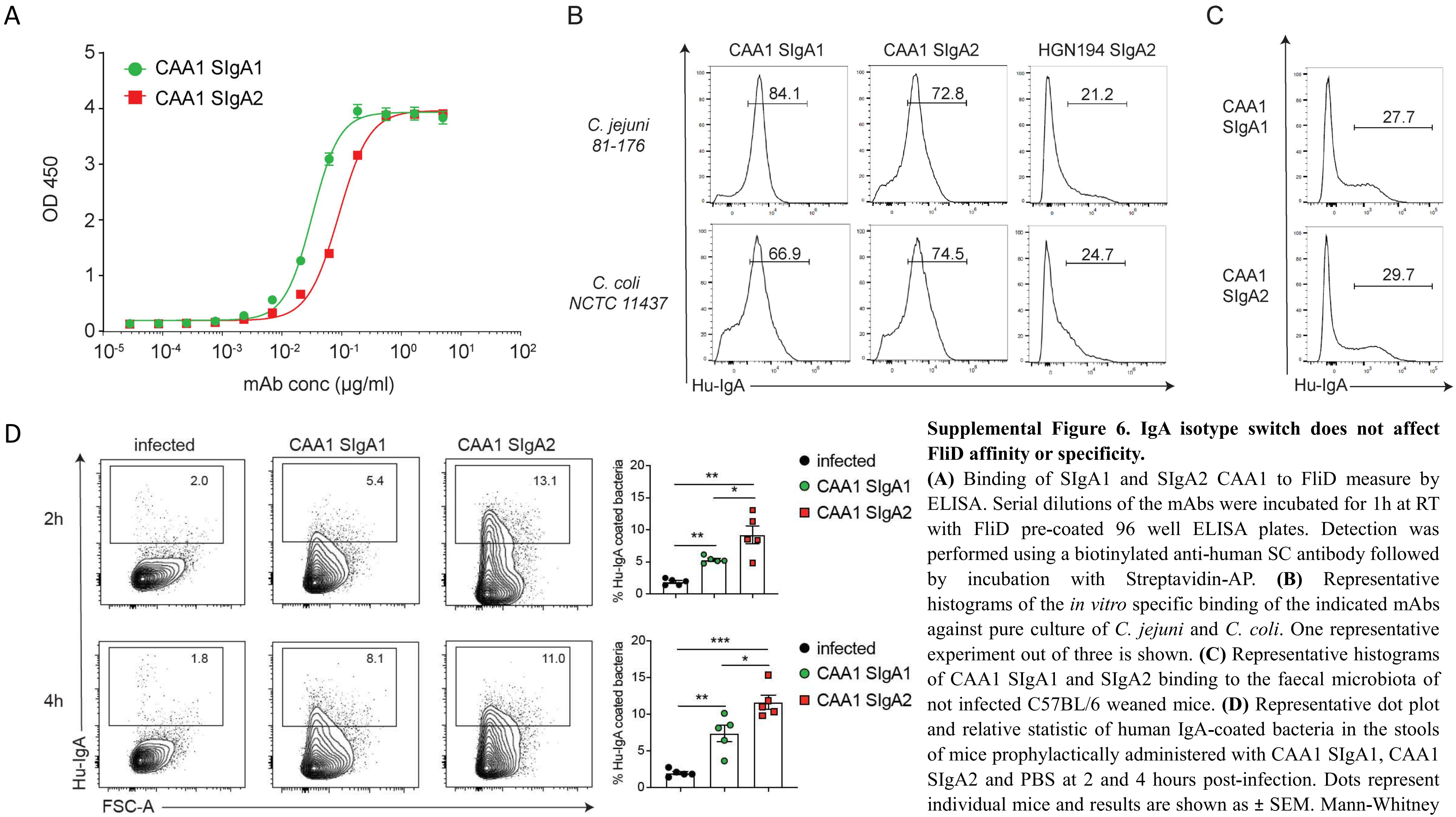

Supplemental Figure 6. IgA isotype switch does not affect FliD affinity or specificity.
(A) Binding of SIgA1 and SIgA2 CAA1 to FliD measure by ELISA. Serial dilutions of the mAbs were incubated for 1h at RT with FliD pre-coated 96 well ELISA plates. Detection was performed using a biotinylated anti-human SC antibody followed by incubation with Streptavidin-AP. **(B)** Representative histograms of the *in vitro* specific binding of the indicated mAbs against pure culture of *C. jejuni* and *C. coli*. One representative experiment out of three is shown. **(C)** Representative histograms of CAA1 SIgA1 and SIgA2 binding to the faecal microbiota of not infected C57BL/6 weaned mice. **(D)** Representative dot plot and relative statistic of human IgA-coated bacteria in the stools of mice prophylactically administered with CAA1 SIgA1, CAA1 SIgA2 and PBS at 2 and 4 hours post-infection. Dots represent individual mice and results are shown as ± SEM. Mann-Whitney test was used. $*p < 0.05$, $**p < 0.01$, $***p < 0.001$.
