# Supplementary material for: Prophylactic Activity of Orally Administered FliD-Reactive Monoclonal SIgA Against Campylobacter Infection
Source: Front Immunol. 2020 Jun 9;11:1011. doi: 10.3389/fimmu.2020.01011 (PMC7296071; doi:10.3389/fimmu.2020.01011)
Supplement: Supplementary file 7 [file Data_Sheet_7.pdf]

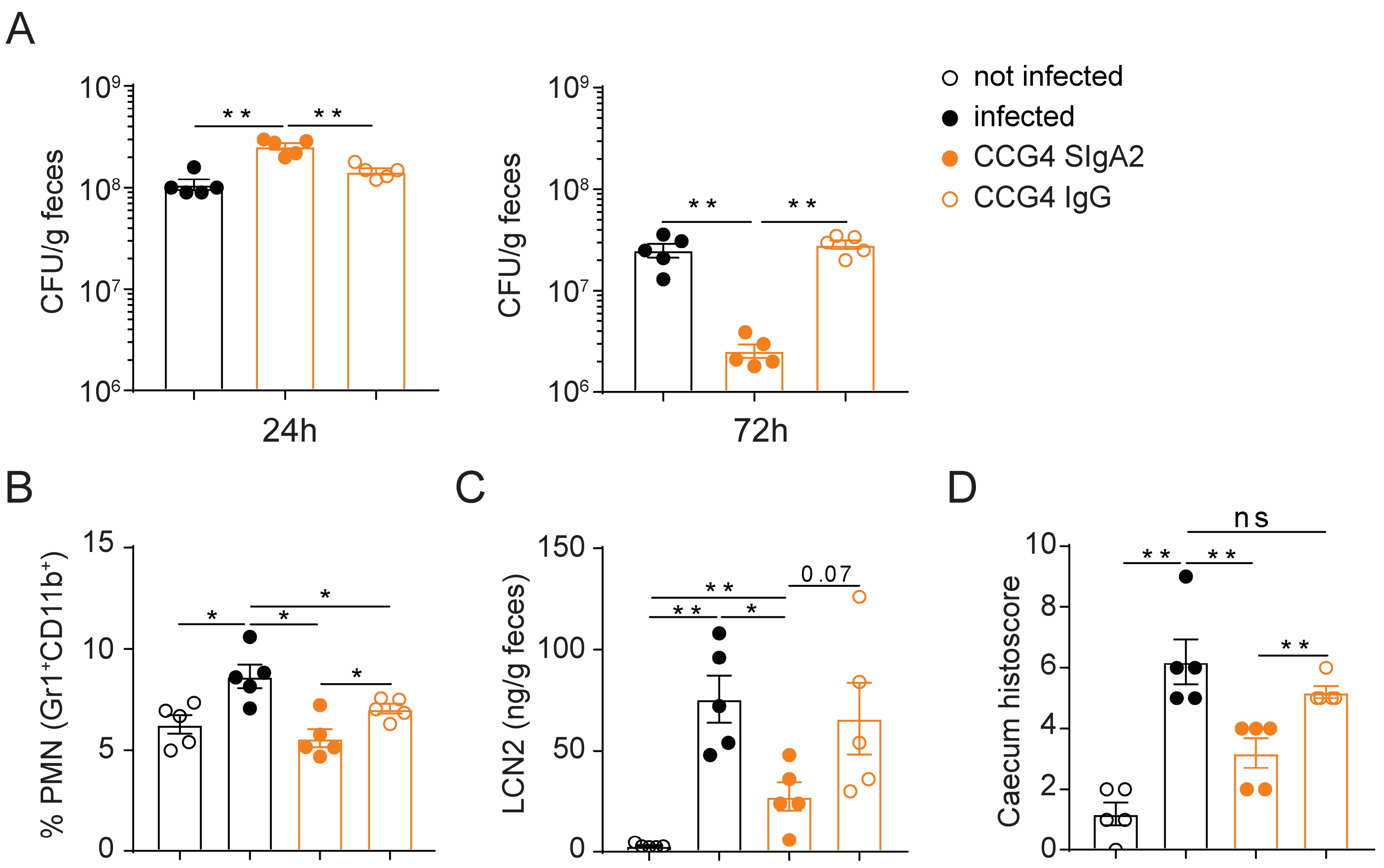


**Supplemental Figure 7. Conversion to IgG reduces CCG4 mAb prophylactic activity against *C. jejuni* infection.**
**(A-D)** 2 hours prior to infection with $10^8$ CFU of C. jejuni, 21-day-old C57BL/6 mice were orally administered via gavage with 200 µg of CCG4 as SIgA2 or IgG1. **(A)** Quantification of the bacterial load (CFU) in the stools of the animals at 24 and 72h post-infection. **(B)** Representative dot plot and relative quantification of polymorphonucleated cells infiltrated in the caecum. **(C)** Quantification of Lipocalin-2 (LCN2) in the stools, and **(D)** statistical analysis of histopathological score in the caecum at 72h post-infection in the different treatment conditions. Dots represent individual mice and results are shown as ± SEM. Mann-Whitney test (A–D) was used. *p < 0.05, **p < 0.01. One representative experiment out of two is shown.
